# Genetic disparities in sleep traits and human capital development: A 25-year study in Finnish population-based cohorts

**DOI:** 10.5271/sjweh.4255

**Published:** 2026-01-01

**Authors:** Aaro Hazak, Katri Kantojärvi, Sonja Sulkava, Merike Kukk, Tuija Jääskeläinen, Veikko Salomaa, Seppo Koskinen, Markus Perola, Tiina Paunio

**Affiliations:** 1 Finnish Institute for Health and Welfare, Department of Public Health, Helsinki, Finland.; 2 University of Helsinki, Department of Psychiatry / SleepWell Research Program, Faculty of Medicine, Helsinki, Finland.; 3 Aalto University, Department of Finance, Espoo, Finland.; 4 Tallinn University of Technology, Department of Economics and Finance, Tallinn, Estonia.; 5 HUS Helsinki University Hospital, Department of Psychiatry, Helsinki, Finland.; 6 HUS Helsinki University Hospital, Department of Clinical Genetics, Helsinki, Finland.; 7 University of Turku, Department of Internal Medicine, Turku, Finland.; 8 University of Helsinki, Research Program for Clinical and Molecular Metabolism, Faculty of Medicine, Helsinki, Finland.

**Keywords:** education, income, insomnia, long sleep, occupation, polygenic index, short sleep, sleep duration

## Abstract

**Objectives:**

Sleep supports cognitive performance and recovery, shaping human capital development through education and workplace knowledge application. This study investigates how polygenic indices (PGI) for insomnia (IPGI), short sleep (SSPGI), long sleep (LSPGI), and sleep duration (SDPGI) are associated with educational attainment, occupational group, and income in the Finnish general population.

**Methods:**

Genetic and socioeconomic registry data were merged with pooled data from six pentennial (1992–2017) cohorts representative of Finnish regional populations aged 25–64 (N=20 121). Regression models assessed associations between sleep trait PGI and human capital outcomes. In extended regression models, phenotypic sleep traits were treated as endogenous variables—potentially influenced by unobserved confounders—and instrumented with their respective PGI to isolate variation attributable to genetic predisposition.

**Results:**

IPGI, SSPGI, and LSPGI were substantially negatively associated with educational attainment (P<0.001) and selection into knowledge work occupational group (P≤0.005). Their negative association with income (P<0.005) primarily operated through pathways involving education and occupational group. Extended regression models confirmed that these PGI validly predicted their respective phenotypic sleep traits, which, when instrumented, were significantly negatively associated with education and belonging to the knowledge work occupational group, supporting causal pathways linking genetic sleep predispositions to human capital outcomes via phenotypic sleep traits. In contrast, SDPGI—an aggregate proxy for genetically distinct short and long sleep traits—was not significantly associated with any human capital outcome.

**Conclusions:**

Genetic predispositions to insomnia, short sleep, and long sleep were robustly and substantially negatively associated with human capital development. These associations may help to clarify how genetic sleep traits relate to outcomes in work and health contexts.

Sleep plays a vital role in cognitive functioning, daily performance, and overall health. Both short and long sleep durations have been linked to cognitive impairment, adverse health outcomes, and increased mortality risk (1–7). Long sleep, often associated with sleep fragmentation or underlying health conditions, may indicate hypersomnia (8–11), while insomnia is also strongly associated with reduced functioning and adverse health outcomes (12–14).

Beyond its health implications, sleep behavior has significant socioeconomic consequences. It is associated with educational attainment, occupational performance, earnings, and overall well-being (15–19). Understanding the role of sleep in human capital development—the enhancement of skills and knowledge for workforce engagement—is essential for addressing inequalities in labor market outcomes and promoting health equity. As the economic landscape shifts toward knowledge-intensive work, adequate sleep becomes increasingly important for sustaining productivity and reducing socioeconomic disparities.

However, the relationship between sleep and economic outcomes is complex and potentially bidirectional. While adequate sleep can enhance cognitive capacity and labor productivity (18, 20), higher wages may reduce sleep due to increased opportunity costs (20, 21). These dynamics introduce concerns of endogeneity, whereby health status, behaviors, and environmental factors may confound observed associations (22–25).

Twin and genomic studies suggest that sleep traits are moderately heritable, with estimates ranging from 38–59% for insomnia and 9–45% for sleep duration (10, 26–31). With the increasing availability of large-scale genome-wide association studies (GWAS), it has become possible to summarize genetic predisposition to complex traits using polygenic indices (PGI). A PGI is a single score that combines information across hundreds of thousands of common genetic variants, each of which individually has a very small effect. These scores are calculated by weighting a person’s genetic variants according to how strongly they were associated with a given trait in an independent GWAS and then summing them into an index.

For sleep, PGI have been developed for insomnia (IPGI), short sleep (SSPGI), long sleep (LSPGI), and overall sleep duration (SDPGI) (10, 26). These traits are highly polygenic, meaning that many genetic variants across the genome contribute to them, each with only modest effect sizes. The biological pathways through which these variants influence habitual sleep remain only partially understood, but PGI allow researchers to capture the cumulative genetic liability to different sleep behaviors at the population level.

The use of PGI has several advantages in epidemiological research. Because genetic variants are determined at conception, PGI are not influenced by later-life environments, health, or behaviors. This makes them useful for addressing concerns about confounding (eg, lifestyle factors that influence both sleep and socioeconomic outcomes) and reverse causality (eg, education or work influencing sleep patterns). At the same time, PGI have important limitations: they reflect only common genetic variants identified in GWAS, do not capture environmental determinants of sleep, and cannot fully separate direct genetic effects from influences such as parental environment (“genetic nurture”) (32, 33). Moreover, pleiotropy—where the same genetic variants affect multiple traits—remains a possibility.

Despite these caveats, PGI provide a valuable tool for studying predisposed variation in sleep traits in large population samples. In the present study, they enabled us to investigate how different dimensions of sleep—insomnia, short and long sleep, and overall sleep duration—relate to education, occupational choices, and income. Sleep duration, often used as an aggregate sleep measure, correlates strongly at the genetic level with short (-0.89) and long sleep (0.68), but only moderately with insomnia (-0.42), and short and long sleep themselves exhibit weak genetic correlation (-0.28) (10, 26). This suggests distinct genetic architectures underlie insomnia, short sleep, and long sleep.

Previous research reports weak-to-moderate genetic correlations between sleep traits and human capital indicators. Insomnia is weakly negatively correlated with education (-0.19) and income (-0.26) (26), while short and long sleep show moderate negative correlations with schooling (-0.35 to -0.36) (10). In contrast, sleep duration shows only weak positive genetic correlations with education (0.13) and income (0.12) (34), reinforcing the need to analyze specific sleep traits separately in relation to socioeconomic outcomes.

This study adopts a comparative approach to assess how polygenic predispositions for insomnia, short and long sleep, and overall sleep duration relate to human capital development. We examine educational attainment, and entry into knowledge work—the occupational group with the highest added value. Additionally, we assess whether these genetic proxies are linked to occupational group selection and income—reflecting labor market returns to skills and effort—beyond their associations through education.

To address the inherent difficulty of inferring causal pathways from PGI to human capital outcomes (32), we apply extended regression models. In these models, self-reported habitual sleep traits—sleep problems, short sleep, long sleep, and overall sleep duration—are treated as endogenous variables and instrumented with their respective PGI: IPGI, SSPGI, LSPGI, and SDPGI. Here, endogeneity refers to the possibility that observed sleep traits are influenced by unmeasured factors that also affect human capital outcomes, potentially biasing associations. Instrumentation uses PGI as independent predictors—fixed at conception and unaffected by later environments—to isolate the variation in sleep traits attributable to genetic predisposition. This approach helps to mitigate potential confounding from environmental factors and reverse causality, allowing us to examine whether the associations between PGI and human capital outcomes operate specifically through habitual sleep-related pathways. It enables us to evaluate (i): whether the PGI effectively predict their corresponding sleep traits in our sample (ii); whether sleep traits, when instrumented with their respective PGI, are significantly associated with human capital outcomes; and (iii) whether the residual variation in sleep traits and human capital outcomes is correlated, potentially indicating shared unobserved determinants.

This study aimed to investigate the associations between polygenic indices for insomnia and sleep duration traits, and human capital outcomes in Finland. Specifically, we focused on educational attainment, occupational groups, and income to better understand how unmodifiable genetic predispositions to sleep traits relate to work, health, and environment.

## Methods

### Study population and data sources

We used pooled genetic and survey data from the Finnish National FINRISK Study (FR; 1992–2012) (35) and the FinHealth 2017 Study (FH) (36), linked with national registry data on education and labor market status. These repeated cross-sectional surveys are representative of the Finnish adult population across multiple regions. The target population consisted of individuals aged 25–64 years. Participants were excluded if they lacked genetic or registry data, resided in Lapland where data were unavailable in most years, or had missing or inconsistent household information. To enhance the accuracy of individual income measurement derived from household data, we further restricted the sample to households with ≤10 members and no more than one other adult besides the survey respondent. These exclusions helped reduce ambiguity regarding the number of earners and income attribution. The final analytic sample included 20 121 participants.

The Finnish Institute for Health and Welfare and/or the Coordinating Ethical Committee of the Helsinki and Uusimaa Hospital District approved the study protocols (approval numbers 38/96, 558/E3/2001, 229/E0/06, 162/13/03/00/2011 and 37/13/03/00/2016). All participants provided written informed consent. The study was conducted in accordance with the Declaration of Helsinki.

### Genetic measures

Genetic data were extracted from blood samples collected during in-person visits. PGI were calculated using the Polygenic Risk Score - Continuous Shrinkage (PRS-CS) method, applying Bayesian regression with continuous shrinkage priors. The European 1000 Genomes Project served as the reference panel. For each participant, the IPGI, SSPGI, LSPGI, and SDPGI were computed as a weighted sum of risk allele counts, with inferred variant effect sizes as weights. The PGI were derived from GWAS conducted using UK Biobank data.

IPGI was based on a GWAS including 109 548 cases and 277 440 controls (26), where insomnia was identified via a validated survey question: *“*Do you have trouble falling asleep at night or do you wake up in the middle of the night?”. While this item captures key symptoms of insomnia (difficulty initiating and maintaining sleep), it does not assess frequency, chronicity or daytime impairment required by common diagnostic criteria, nor does it capture heterogeneity in insomnia subtypes (37). Thus, the IPGI reflects genetic liability to insomnia-related symptoms at the population level, rather than strictly defined clinical insomnia disorder.

SSPGI, LSPGI, and SDPGI were based on GWAS data from 446 118 individuals, based on self-reported habitual sleep duration (<7 hours=short sleep, ≥9 hours= long sleep), referring to total 24-hour sleep including naps, with no differentiation between workdays and free days, and supported by accelerometer data from 85 499 participants (10). These indices capture distinct but related aspects of sleep behavior. SDPGI reflects genetic liability to continuous variation in habitual sleep duration across the population, while the SSPGI and LSPGI capture genetic predisposition toward the extreme phenotypes of short (<7 hours) and long (≥9 hours) sleep, respectively. Although correlated [supplementary materials (www.sjweh.fi/article/4255) table S1], these PGI are not interchangeable: SDPGI indexes general variation in sleep duration, whereas SSPGI and LSPGI represent specific sleep phenotypes that may have unique consequences for health and socio-economic outcomes.

Each PGI comprised approximately one million variants and was standardized to have a mean of zero and a standard deviation (SD) of one, using R 3.6.0 software. Full genotyping details are provided in Hazak et al (38).

It is important to note that PGI do not directly measure sleep parameters but aggregate common genetic variants statistically associated with sleep traits in large GWAS. While some variants lie near genes of potential biological relevance, most have not been functionally characterized. In this study, PGI are therefore used as genetic proxies for sleep behaviors—capturing inherited propensities for insomnia, short sleep, long sleep, or sleep duration—rather than pinpointing causal biological mechanisms.

### Phenotypic sleep traits and validation of genetic proxies

To validate the PGI within our sample, we used two survey-based sleep measures. The first included self-reported habitual sleep duration, available from FR 2007 and 2012 and FH 2017 cohorts (N=8148). Sleep hours were categorized as short sleep (<7 hours; N=1242), long sleep (≥9 hours; N=950), and intermediate sleep (≥7–<9 hours; N=5956). Wilcoxon rank-sum tests comparing SSPGI and SDPGI distributions among the short sleep and combined long/intermediate sleep categories, and LSPGI and SDPGI distributions among the long sleep and combined short/intermediate sleep categories, revealed highly significant differences (P<0.001), confirming that SSPGI, LSPGI, and SDPGI effectively distinguish between the respective sleep duration categories.

The second measure, sleep problems, was available across all study years (N=19 965). It was based on the question, “Do you have trouble sleeping?” (FR) or “Over the past month, how often have you had trouble sleeping?” (FH), rated on a Likert scale: “often,” “sometimes,” and “not at all.” Wilcoxon rank-sum tests comparing IPGI distributions between the “often” and combined “sometimes/not at all” groups showed highly significant differences (P<0.001), validating the ability of IPGI to differentiate individuals with self-reported sleep problems.

Additionally, extended regression models (described below) were used to further confirm the predictive validity of each PGI for its respective phenotypic sleep trait within our study sample.

### Outcome measures

Educational attainment and labor market status were derived from registry data compiled by Statistics Finland. Labor market status referred to employment status and occupational group at the beginning of each study year (or the previous year for the 1992–2002 cohorts). Occupational groups were derived from Statistics Finland’s national administrative registry classification of socio-economic groups, which follows the United Nations Economic Commission for Europe (UNECE, 2010) statistical recommendations for population censuses (Statistics Finland, 2018). These categories are broadly comparable to the International Standard Classification of Occupations (ISCO-08) (International Labor Organization, 2012). In our study, the “physical work” group comprises manual workers, aligning with ISCO major groups 6–9; “office work” corresponds to lower-level administrative and clerical occupations in ISCO groups 4–5; and “knowledge work” reflects upper-level administrative, managerial, professional and related occupations broadly consistent with ISCO groups 1–3. The term “knowledge work” is adopted from prior literature (39, 40), which emphasizes the importance of cognitive, analytical, and problem-solving tasks as defining characteristics of modern professional and managerial roles.

Income was self-reported and recorded in household income bands. We calculated midpoint values for each band, adjusted income based on the number of adult household members (one or two), and created income quintiles for comparability across survey years. As assortative mating patterns are pronounced in Nordic countries (41), the adjusted household income measure provides a useful proxy for individual earnings.

### Control variables

Control variables obtained from registry data included gender, age, birth cohort, and study year. To adjust for population stratification, we included the first three genetic principal components (PC1–PC3), consistent with standard practice in Finnish genetic epidemiology. Given the high degree of genetic homogeneity in the Finnish population, PC1–PC3 capture the principal axes of genetic variation. Limiting the adjustment to these three components balances the need to control for ancestry-related structure while avoiding overfitting or reduced model precision that may arise from the inclusion of higher-order principal components with limited explanatory value.

### Descriptive statistics

Table 1 presents key descriptive statistics, with additional details provided in supplementary table S2. As shown in supplementary table S2, individuals in the lowest decile of the short sleep PGI (mean SSPGI=-1.77) have, on average, a LSPGI score (0.04) only slightly above the sample mean (0.00). Conversely, those in the lowest decile of the long sleep PGI (mean LSPGI=-1.76) have, on average, a SSPGI score (0.06) also slightly above the sample mean (0.00). These patterns highlight the largely distinct genetic architectures underlying short and long sleep in our sample.

**Table 1:** Descriptive statistics of the pooled Finnish National FINRISK (FR) Study 1992–2007 (35) and the FinHealth (FH) 2017 sample. [SD=standard deviation; IPGI=insomnia polygenic index; SSPGI=short sleep polygenic index; LSPGI=long sleep polygenic index; SDPGI=sleep duration polygenic index.]

		All (N=20 121)					Male (N=9248)					Female (N=10 873)			
		Mean	%	Min-Max	SD		Mean	%	Min-Max	SD		Mean	%	Min-Max	SD
IPGI		-0.001		-3.72–4.28	1.002		-0.004		-3.72–4.09	0.999		0.001		-3.64–4.28	1.005
SSPGI		0.001		-4.02–4.03	0.998		-0.011		-4.02–3.58	0.990		0.011		-3.83–4.03	1.004
LSPGI		0.002		-3.96–4.09	0.999		-0.021		-3.96–3.82	0.994		0.022		-3.48–4.09	1.003
SDPGI		-0.003		-3.87–4.32	1.004		-0.004		-3.87–3.74	1.000		-0.002		-3.80–4.32	1.008
Sleep hours ^a^		7.530		0.00–16.00	1.206		7.445		0.00–16.00	1.250		7.605		0.00–14.00	1.160
Short sleep (yes=1) ^a^			15					17					14		
Long sleep (yes=1) ^a^			12					10					13		
Sleep problems (often=1) ^b^			8					7					10		
Education															
	Primary (ref)		20					22					18		
	Secondary		42					45					40		
	Higher		38					33					42		
Labor market status ^c^															
	Non-employed		25					26					23		
	Self-employed		9					12					7		
	Physical work		21					28					16		
	Office work		28					17					38		
	Knowledge work		17					18					17		
Income quintile															
	Lowest		22 ^d^					21					22		
	Lower		20					20					21		
	Medium		19 ^d^					18					20		
	Higher		20					20					19		
	Highest		20					21					18		
Gender (male=0; ref)			46					100					0		
Age		44.9		25–64	11.7		45.3		25–64	11.6		44.5		25–64	11.7

^a^ Sleep hours were available only for FR 2007 and 2012, and FH 2017 cohorts (N=8148). These values were self-reported and thus subject to misreporting biases, including 0 values. However, outliers were not ample, as illustrated in the histogram in supplementary figure S2. The self-reported sleep hours were used to construct binary indicators for short sleep (yes=1 if <7 hours) and long sleep (yes=1 if ≥9 hours). ^b^ Sleep problems were assessed using responses to the survey question: “Do you have trouble sleeping?” (FR) or “Over the past month, how often have you had trouble sleeping?” (FH). Responses were harmonised and dichotomised into two categories: “Often” (indicating sleep problems) and a combined reference category of “Sometimes” and “Not at all”. ^c^ Encompasses both employment status (designated as category 1 “non-employed”) and occupational groups (categories 2-5). Specifically, category 2, “self-employed,” includes individuals classified as “self-employed persons”; category 3, “physical work,” comprises those classified as “manual workers”; category 4, “office work,” includes individuals classified as “lower-level employees with administrative and clerical occupations”; and category 5, “knowledge work,” encompasses those classified as “upper-level employees with administrative, managerial, professional, and related occupations” according to the Statistics Finland registry data. ^d^ Some income quintile sizes in the full sample differ slightly from 20% because income was measured in range categories in the FR and FH surveys, which does not allow for allocation of participants into income groups of exactly 20%.

Histograms of the IPGI, SSPGI, LSPGI and SDPGI by gender have been presented in supplementary figure S1, and pairwise linear correlation matrix in supplementary table S1.

### Statistical analysis

To examine associations between genetic predisposition for sleep traits and sequential human capital outcomes—educational attainment, labor market status, and income—we estimated a series of regression models using each of the four PGI as the primary explanatory variable. PGI were specified in multiple formats as: continuous linear variables, non-linear polynomials, and decile-based comparisons. Ordered probit (oprobit) models were used for education and income, while probit models were employed for binary labor market statuses, particularly the probability of belonging to the knowledge work occupational group.

All models adjusted for gender, birth cohort dummies (categorized by decades), and PC1–PC3. Models for labor market status and income additionally included study year dummies and age (modelled as both linear and squared terms). Since genetic predispositions for sleep traits are stable across the life course, and both educational and occupational outcomes are relatively fixed in adulthood, the repeated cross-sectional nature of our data does not materially limit interpretation of the results.

To examine gender-specific patterns—given observed disparities in education, occupational segregation, and income (table 1)—we included PGI-gender interaction terms and estimated gender-stratified models.

Given education and income disparities across occupational groups (supplementary figure S3), we further assessed whether associations between sleep-related PGI and occupational or income outcomes were mediated through education, and conducted subgroup analyses restricted to individuals with higher education. Within this more homogeneous educational group, we evaluated whether PGI continued to be associated with occupational group and income, likely reflecting labor market valuation of sleep-related traits beyond educational attainment. Additional models were estimated for individuals with higher education in the knowledge work occupational group to further isolate associations between the PGI and income within that relatively uniform career segment.

Given the strong patterning of phenotypic sleep traits by education, occupational group, and income (supplementary figure S4), we sought to better understand whether the observed associations entail potential causal pathways from genetic proxies for sleep behaviors to human capital outcomes via phenotypic sleep traits. We applied Extended Regression Models (ERM; StataCorp LLC, 2023) employing maximum likelihood estimation to simultaneously estimate a system of two equations (i): a first-stage model predicting a self-reported sleep trait—sleep problems (probit), short sleep (probit), long sleep (probit), or sleep hours (linear)—instrumented by its respective PGI (IPGI, SSPGI, LSPGI, or SDPGI); and (ii) a second-stage main equation predicting a human capital outcome—educational attainment (ordered probit), belonging to the knowledge work occupational group (probit), or income (ordered probit)—that includes the phenotypic sleep trait as an endogenous explanatory variable that may be influenced by unobserved confounders. The same covariates were included in both equations: gender, age (linear and squared), PC1–PC3, birth cohort, and study year dummies. The PGI was included only in the first-stage sleep trait equation, serving as the instrument—an independent predictor. Instrumenting phenotypic sleep traits in this way helps isolate the variation attributable to genetic predisposition, excluding other pathways through which sleep traits might otherwise influence human capital outcomes. Moreover, this approach helps to exclude potential reverse pathways from human capital outcomes to sleep traits, such as those arising from opportunity costs (20, 21).

All models used robust standard errors (SE) to account for heteroscedasticity, non-normality, and clustering. Average marginal effects are reported as percentage point differences relative to a reference category. Statistical significance was defined as P<0.005, and findings at P<0.05 were considered suggestive, following the recommendations by Benjamin et al (42), and given that each model tested a distinct study question without exploratory analyses in subsamples or multiple comparisons, no additional multiple-testing correction was applied. All analyses were conducted in STATA 18 (StataCorp LLC, 2023), ensuring methodological consistency with our group’s other studies on genetic predictors of brain health and socioeconomic outcomes (38, 43).

## Results

### Genetic disparities in sleep traits and education

**Figure 1:**
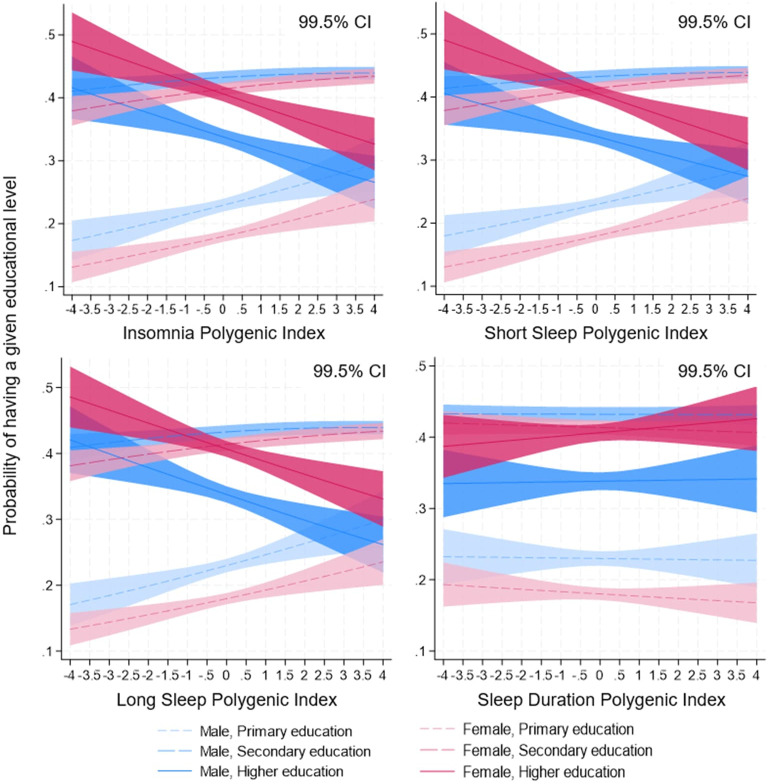
Polygenic indices (PGI) for insomnia (IPGI, upper left), short sleep (SSPGI, upper right), long sleep (LSPGI, lower left) and sleep duration (SDPGI, lower right) and probabilities of having a given educational level in the pooled 1992–2017 sample (N=20 121) with 99.5% confidence interval (CI). Note: figure presenting predicted probabilities from the models in table 2 incorporating gender interaction with IPGI (26), SSPGI (10), LSPGI (10), and SDPGI (10).

Our findings revealed a consistent negative association between educational attainment and three sleep-related polygenic indices: IPGI, SSPGI, and LSPGI (P<0.001; table 2; predicted probabilities in figure 1). These associations were robust across genders (table 2; supplementary table S3) and showed no evidence of non-linear relationships (supplementary table S4). In contrast, SDPGI was not significantly associated with educational attainment under either linear or non-linear specifications (table 2; supplementary tables S3 and S4).

**Table 2:** Coefficient estimates from ordered probit regression models of **education** in the pooled 1992–2017 sample. [SE=robust standard errors; PGI=polygenic indices; IPGI=insomnia PGI;SSGI=short sleep PGI; LSPGI=long sleep PGI; SDPGI=sleep duration PGI.]

		All N=20 121										
		IPGI			SSPGI			LSPGI			SDPGI	
		Coefficient	SE		Coefficient	SE		Coefficient	SE		Coefficient	SE
Variables												
	PGI	-0.056 ***	0.012		-0.049 ***	0.012		-0.059 ***	0.012		0.002	0.012
	Female	0.196 ***	0.016		0.197 ***	0.016		0.198 ***	0.016		0.196 ***	0.016
	PGI # female	-0.002	0.016		-0.009	0.016		0.005	0.016		0.011	0.016
	PC1	9.206 ***	1.142		9.614 ***	1.145		9.127 ***	1.142		9.179 ***	1.148
	PC2	-4.078 ***	1.163		-3.981 ***	1.163		-4.138 ***	1.163		-4.177 ***	1.164
	PC3	0.117	1.099		0.103	1.097		0.232	1.097		-0.083	1.097
	Birth cohort dummies	Yes			Yes			Yes			Yes	
	Cut: primary/secondary education	-0.824 ***	0.019		-0.825 ***	0.019		-0.824 ***	0.019		-0.824 ***	0.019
	Cut: secondary/higher education	0.447 ***	0.018		0.445 ***	0.018		0.447 ***	0.018		0.445 ***	0.018
Pseudo-R^2^		0.071			0.071			0.071			0.070	
p(χ^2^)		***			***			***			***	

*** P < 0.001.

Ordered probit regression models utilizing PGI decile comparisons supported these results. Compared to individuals in the lowest decile, those in the top decile of IPGI, SSPGI, and LSPGI were 8.6, 7.2, and 6.2 percentage points less likely to attain higher education, respectively [all P<0.001; 99.5% confidence intervals (CI) 5.1–12.1, 3.6–10.8, 2.7–9.7 percentage points; supplementary tables S5 and S6]. Considering the baseline probability of higher education in the sample is 38% (table 1), these differences are substantial.

Extended regression models further validated these findings. IPGI was a suggestive predictor of perceived sleep problems (P=0.02), while SSPGI, LSPGI, and SDPGI significantly predicted their respective phenotypic sleep traits (P<0.001; supplementary tables S7 and S8). In turn, these sleep traits, when instrumented with their respective PGI, were significantly negatively associated with educational attainment (P<0.001; supplementary table S7), except for sleep hours, which showed a suggestive positive association (P<0.05; supplementary table S8).

The extended regression model residuals revealed a statistically significant but weak positive correlation between the unobserved components of short sleep and education (r=0.30, P<0.001), and a suggestive weak positive correlation between long sleep and education (r=0.27, P=0.01; supplementary table S7), suggesting the presence of potential shared latent factors beyond genetic predispositions.

### Genetic disparities in sleep traits and knowledge work

**Figure 2:**
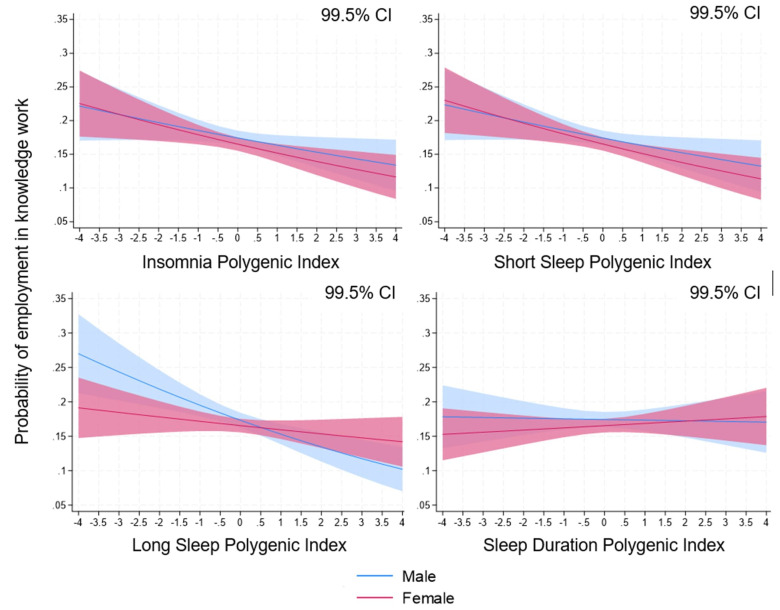
Polygenic indices (PGI) for insomnia (IPGI, upper left), short sleep (SSPGI, upper right), long sleep (LSPGI, lower left) and sleep duration (SDPGI, lower right) and probabilities of belonging to the knowledge work occupational group in the pooled 1992–2017 sample (N=19 707) with 99.5% confidence interval (CI). Note: figure presenting predicted probabilities from the models in table 2 incorporating gender interaction with IPGI (26), SSPGI (10), LSPGI (10), and SDPGI (10).

IPGI, SSPGI, and LSPGI were each negatively associated with the likelihood of being employed in the knowledge work occupational group (P≤0.005; table 3; predicted probabilities in figure 2). For LSPGI, the association was significant only among males (table 3; supplementary table S9). No other statistically significant associations were observed for alternative labor market statuses, except that SSPGI and LSPGI were linked to greater likelihood of employment in the physical work occupational group, offsetting their negative associations with knowledge work (supplementary tables S10–S13). SDPGI showed no meaningful associations with labor market status, and no evidence of non-linear associations between any PGI and labor market status was detected (supplementary tables S10–S13).

**Table 3:** Coefficient estimates from probit models of belonging to the **knowledge work occupational group** in the pooled 1992–2017 sample. [SE=robust standard errors; PGI=polygenic indices; IPGI=insomnia PGI;SSGI=short sleep PGI; LSPGI=long sleep PGI; SDPGI=sleep duration PGI.].

		All N=19 707										
		IPGI			SSPGI			LSPGI			SDPGI	
		Coefficient	SE		Coefficient	SE		Coefficient	SE		Coefficient	SE
Variables												
	PGI	-0.044 **	0.016		-0.044 **	0.016		-0.086 ***	0.016		-0.006	0.016
	Female	-0.037	0.022		-0.036	0.022		-0.031	0.022		-0.036	0.021
	PGI # Female	-0.015	0.022		-0.016	0.021		0.061 **	0.021		0.019	0.021
	Age	0.128 ***	0.010		0.128 ***	0.010		0.128 ***	0.010		0.127 ***	0.010
	Age squared	-0.002 ***	0.000		-0.002 ***	0.000		-0.002 ***	0.000		-0.002 ***	0.000
	PC1	16.627 ***	1.472		17.000 ***	1.478		16.528 ***	1.472		16.551 ***	1.478
	PC2	-1.540	1.438		-1.452	1.436		-1.615	1.436		-1.666	1.436
	PC3	-2.452	1.387		-2.457	1.386		-2.379	1.386		-2.641	1.386
	Year dummies	Yes			Yes			Yes			Yes	
	Birth cohort dummies	Yes			Yes			Yes			Yes	
	Constant	-3.553 ***	0.250		-3.556 ***	0.250		-3.566 ***	0.250		-3.542 ***	0.250
Pseudo-R_2_		0.036			0.036			0.037			0.035	
p(χ^2^)		***			***			***			***	

** P<0.005. *** P<0.001.

Decile-based results reinforced these patterns. Relative to the bottom decile, those in the top decile of IPGI, SSPGI, and LSPGI (males only) were 4.4, 5.6, and 8.9 percentage points less likely, respectively, to be employed in the knowledge work occupational group (all P<0.001; 99.5% CI 1.0–7.8, 2.3–8.9, 4.1–13.8 percentage points; supplementary tables S14 and S15). With only 17% of the sample in the knowledge work occupational group (table 1), these differences are substantial.

Within the higher education subgroup (N=7456), probit models showed a statistically significant negative association between LSPGI and belonging to the knowledge work occupational group (P=0.004), and a suggestive negative association for SSPGI (P=0.045; supplementary table S16). IPGI and SDPGI were not significantly associated in this model, indicating that the associations of LSPGI and SSPGI with knowledge work employment may operate through pathways beyond educational attainment (supplementary table S16).

Extended regression models of employment in the knowledge work occupational group yielded consistent results. SSPGI, LSPGI, and SDPGI significantly predicted their corresponding phenotypic sleep traits (P<0.001), while IPGI was a suggestive predictor of sleep problems (P=0.02; supplementary tables S7 and S8). These sleep traits, when instrumented with their respective PGI, were significantly negatively associated with belonging to the knowledge work occupational group (P<0.001; supplementary table S7), except for sleep hours—instrumented by SDPGI—which showed no significant relationship (supplementary table S8).

Residual correlations from the extended regression models indicated moderate and statistically significant positive associations between unobserved factors affecting knowledge work employment, and both short sleep (r=0.52, P=0.003), and long sleep (r=0.72, P<0.001). A suggestive moderate positive correlation was also observed between knowledge work and sleep problems (r=0.57, P=0.01; supplementary table S7), indicating potential shared latent determinants beyond genetic predispositions.

### Genetic disparities in sleep traits and income

Ordered probit models showed that IPGI, SSPGI, and LSPGI were each negatively associated with income quintile (P<0.005; supplementary table S17).

Among those with higher education (N=7498), LSPGI (particularly among males) and SSPGI (particularly among females) showed suggestive negative associations with income (P<0.05; supplementary table S18). However, when models were restricted to knowledge workers with higher education (N=2910)—where educational background is homogeneous—no significant associations were observed between any PGI and income (supplementary table S19). This suggests that income-related associations for these PGI are primarily mediated through earlier selection processes into education and occupational group.

Extended regression models of income aligned with the main results. IPGI, SSPGI, and LSPGI each predicted of their respective phenotypic sleep traits (supplementary table S7). Among the phenotypic traits, sleep problems—instrumented by IPGI—were significantly negatively associated with income (P<0.001), and short sleep—instrumented by SSPGI—showed a suggestive negative association (P=0.007; supplementary table S7). Long sleep—instrumented by LSPGI—showed no significant association (supplementary table S7). Residual correlations between sleep traits and income were not statistically significant.

SDPGI showed no significant associations with income in any of the models (supplementary table S20 and S8).

## Discussion

This study examined how genetic predispositions for insomnia (IPGI), short sleep (SSPGI), long sleep (LSPGI), and overall sleep duration (SDPGI) relate to human capital development—specifically educational attainment, employment in the knowledge work occupational group, and income—using data from 20 121 working-age Finnish adults between 1992 and 2017.

### Sleep-related genetic predispositions and education

IPGI, SSPGI, and LSPGI were consistently associated with lower educational attainment. Extended regression models confirmed that these PGI significantly predicted their respective phenotypic sleep traits, which, when instrumented with their respective PGI, were negatively associated with educational outcomes. The weaker association between IPGI and its phenotypic proxy may reflect the less precise alignment between the survey-based sleep problem indicator and the GWAS-derived insomnia phenotype. Nonetheless, these results reinforce earlier evidence linking atypical sleep patterns to lower academic performance (15), while demonstrating that such associations have partially a genetic basis.

The weak genetic correlation between SSPGI and LSPGI (-0.28) (10), along with similarly weak correlations between SSPGI and LSPGI, and IPGI and LSPGI, as well as the moderate correlation between IPGI and SSPGI observed in our sample (supplementary table S1), support the interpretation that these PGI reflect distinct pathways. All three PGI showed similarly negative associations with educational attainment, suggesting that different genetic liabilities at opposite ends of the sleep spectrum may lead to comparable disadvantages in educational outcomes. In contrast, SDPGI was not associated with educational outcomes—likely because it aggregates the counterbalancing effects of the genetically distinct short and long sleep components. These findings extend recent research that showed only weak genetic correlations between overall sleep duration and educational attainment (34). Our findings support the hypothesis that genetic risks associated with sleep disorders—often reflecting underlying somatic or neuropsychiatric conditions (9–11)—rather than overall sleep duration, contribute to educational disparities. However, the PGI may not solely capture predisposition to short sleep, long sleep, or insomnia symptoms; these could also reflect overlapping mechanisms such as hyperarousal and pleiotropic pathways shared with somatic and mental health conditions. This limits the specificity of the measure and suggests that part of its association with educational and labor market outcomes may operate through these related mechanisms rather than sleep traits per se. Future studies integrating polygenic indices with physiological and clinical assessments could help disentangle these pathways more precisely.

The observed positive correlations between the unobserved components of education and both short and long sleep in the extended regression models suggest the presence of shared latent influences beyond genetic predispositions—possibly reflecting neurodevelopmental or early-life environmental factors. Future research should explore how sleep-related genetic vulnerabilities interact with environmental factors such as family environment, schooling conditions, and social support systems (44) in shaping both habitual sleep traits, and educational and subsequent career trajectories.

Our measure of education was restricted to the highest completed degree level, which does not account for academic performance indicators such as grades, honors or field of study. Consequently, while we observed associations between genetic proxies of sleep behaviors and the likelihood of attaining higher education, these results do not address potential differences in performance within educational levels.

### Sleep-related genetic predispositions and knowledge work

IPGI, SSPGI, and LSPGI were also negatively associated with employment in the knowledge work occupational group, even after accounting for educational attainment. This implies that sleep-related traits may be associated with occupational group not only through formal education but also through other channels—such as sustained attention, cognitive performance, or emotional regulation—which are particularly valued in knowledge-intensive occupations. Again, SDPGI was unrelated to occupational groups, reinforcing the view that overall sleep duration lacks directional predictive power.

Extended regression models confirmed that phenotypic sleep traits, instrumented by their respective PGI, were significantly associated with lower likelihood of belonging to the knowledge work occupational group. These findings help to mitigate concerns about reverse causality or confounding, highlighting the importance of genetic sleep predispositions in shaping occupational outcomes via habitual sleep traits. As economies shift toward knowledge-driven sectors, the implications of sleep-related vulnerabilities may widen, particularly in high-skill job environments, emphasizing the societal costs of suboptimal sleep duration (45).

### Sleep-related genetic predispositions and income

Higher IPGI, SSPGI, and LSPGI were associated with lower income, though these associations appeared to be largely mediated by earlier educational and occupational selection. Among those with higher education belonging to the knowledge work occupational group, these PGI were no longer associated with income, suggesting that the earnings associations of sleep-related genetic predispositions operate primarily through upstream pathways. SDPGI showed no significant relationship with income.

### Inclusion and occupational health implications

Although genetic predispositions themselves are not modifiable, their associations with educational and occupational outcomes are (32). Policies and practices that promote sleep health, support educational inclusion, and enable flexible workplace conditions may help buffer the disadvantages faced by individuals with greater vulnerability to sleep problems. As knowledge work and productivity enhancement become increasingly central to economic development (40), understanding how inherent sleep traits relate to access to and success in these occupations can inform efforts for socioeconomic inclusion. Importantly, genetic predispositions do not determine individual outcomes: health, occupational, environmental, institutional, and policy contexts play a decisive role in shaping career and income trajectories (32). Inclusive policies and practices that recognize and accommodate diversity in sleep traits can therefore contribute to fairer opportunities and improved outcomes in education, work, and well-being. From an occupational health and safety perspective, the findings suggest that genetic predispositions to atypical sleep traits may contribute to inequalities in meeting job demands and sleep-related health risks across occupational groups—areas that warrant further investigation.

### Study limitations

While the FR and FH studies are regionally representative, some degree of selection bias may be present due to voluntary participation in genetic sampling. Furthermore, the PGI were constructed using GWAS data from UK populations, which may limit their transferability to the Finnish context. As the sample is limited to individuals of Finnish ancestry, generalizability to other genetic, cultural, or socioeconomic contexts is constrained. A limitation of the sleep duration measures is that both the underlying GWAS (10) and the FH and FR surveys relied on a single self-reported estimate of total 24-hour sleep, without differentiating between workdays and free days. As many individuals shorten their sleep on workdays and extend it on weekends, this approach may introduce misclassification of genetic predisposition to habitual short or long sleep. Consequently, the PGI may capture both endogenous sleep regulation and behavioral adaptations to social or work schedules. Such measurement limitations are likely to introduce random error, which would attenuate rather than inflate our estimates, suggesting that the true associations may be stronger. Nevertheless, the study provides important new evidence on how genetic predispositions for sleep traits relate to education and labor market outcomes.

### Concluding remarks

Genetic predispositions to insomnia, short sleep, and long sleep were consistently associated with lower educational attainment and reduced likelihood of belonging to the knowledge work occupational group in Finnish population-based cohorts. Extended regression models reinforced these associations and confirmed the predictive utility of polygenic indices for phenotypic sleep traits. By treating habitual sleep traits as endogenous and instrumenting them with their respective polygenic indices, we were able to isolate genetic variation and strengthen causal interpretation. These findings suggest that inherited variation in sleep traits may contribute to inequalities in human capital development. Future research should examine replication in other populations and consider potential pathways linking sleep genetics to work, health and socioeconomic outcomes.

## Supplementary material

Supplementary material

## Data Availability

The FR and FH data that support the findings of this study are available from the Finnish Institute for Health and Welfare subject to permission.
