# Supplementary material for: Genetic disparities in sleep traits and human capital development: A 25-year study in Finnish population-based cohorts
Source: Scand J Work Environ Health. 2025 Dec 27;52(1):63–74. doi: 10.5271/sjweh.4255 (PMC12782571; doi:10.5271/sjweh.4255)
Supplement: Supplementary material [file SJWEH-52-63-S001.pdf]

Genetic disparities in sleep traits and human capital development: A 25-year study in Finnish population-based cohorts[1]

by Aaro Hazak, PhD,[2] Katri Kantojärvi, PhD, Sonja Sulkava, PhD, Merike Kukk, PhD, Tuija Jääskeläinen, PhD, Veikko Salomaa, PhD, Seppo Koskinen, PhD, Markus Perola, PhD, Tiina Paunio, PhD

Hazak A, Kantojärvi K, Sulkava S, Kukk M, Jääskeläinen T, Salomaa V, Perola M, Paunio T. Genetic disparities in sleep traits and human capital development: A 25-year study in Finnish population-based cohorts. *Scand J Work Environ Health* – online first.

1. Supplementary material
2. Correspondence to: Aaro Hazak, SleepWell Research Program, Faculty of Medicine, University of Helsinki, Välskärinkatu 12, Helsinki 00014, Finland. [E-mail: aaro.hazak@helsinki.fi]

Figure S1 Histograms of IPGI, SSPGI, LSPGI and SDPGI in the pooled 1992-2017 sample

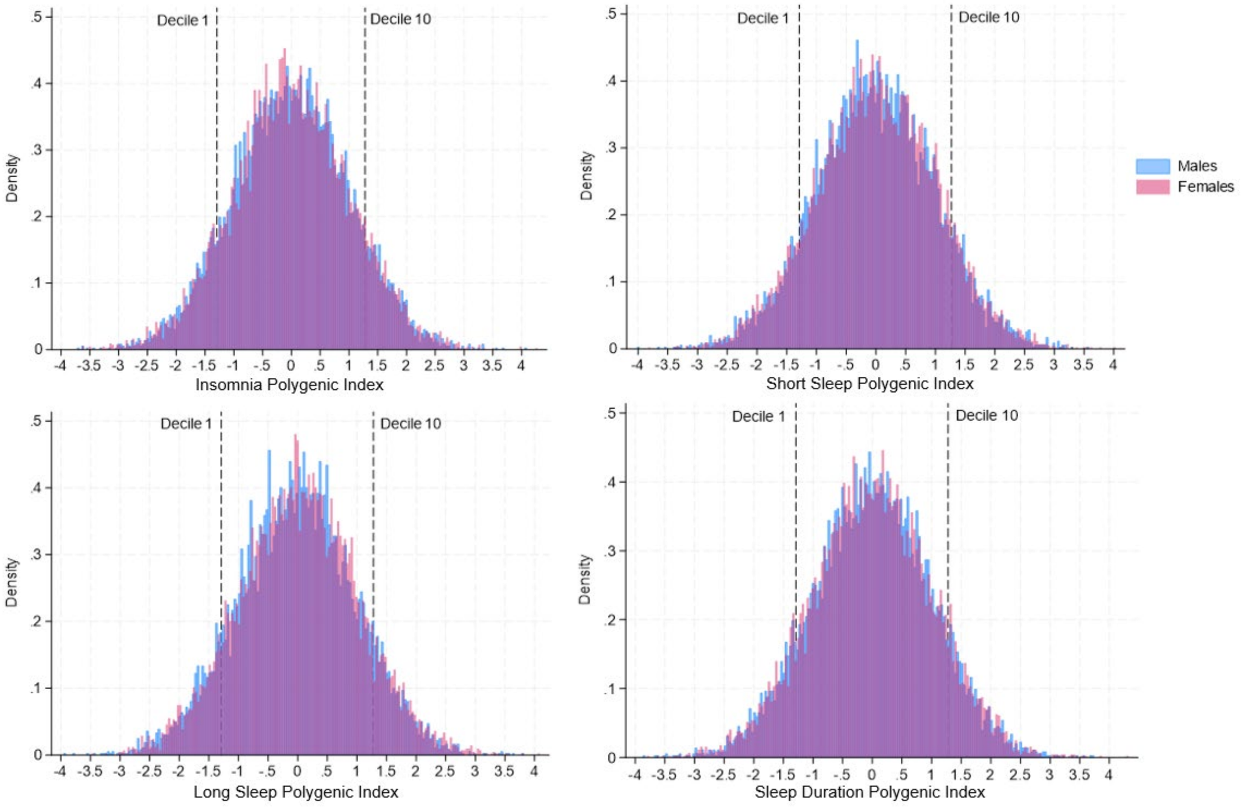

Note: Polygenic indices (PGI) for insomnia (IPGI) (26), short sleep (SSPGI) (10), long sleep (LSPGI) (10), and sleep duration (SDPGI) (10)

Figure S2 Histograms of sleep hours in the pooled 2007-2017 sample

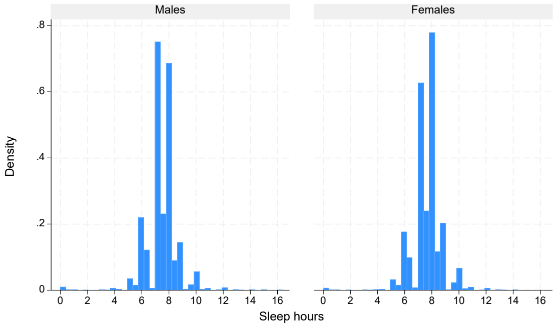

Figure S3 Education (left) and income (right) by labour market status and gender in the pooled 1992-2017 sample

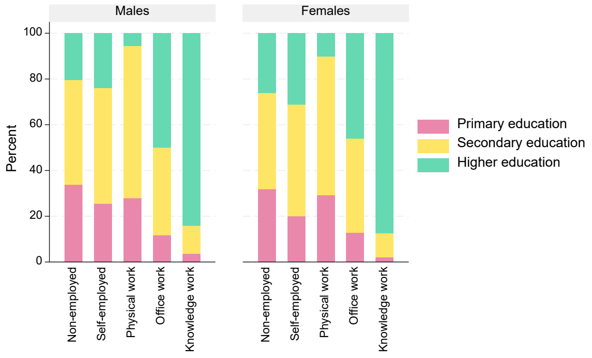

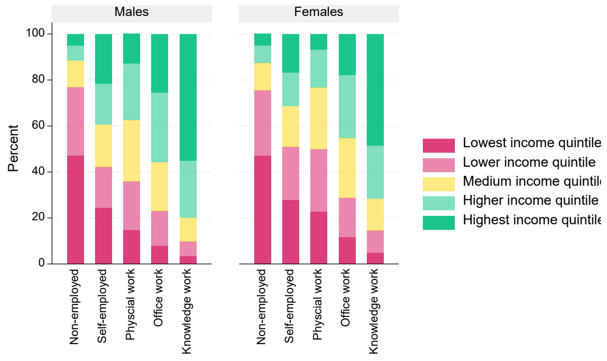

Figure S4 Prevalence of sleep problems, short sleep, and long sleep by education, labour market status, and income categories, stratified by gender in the pooled 1992-2017 sample

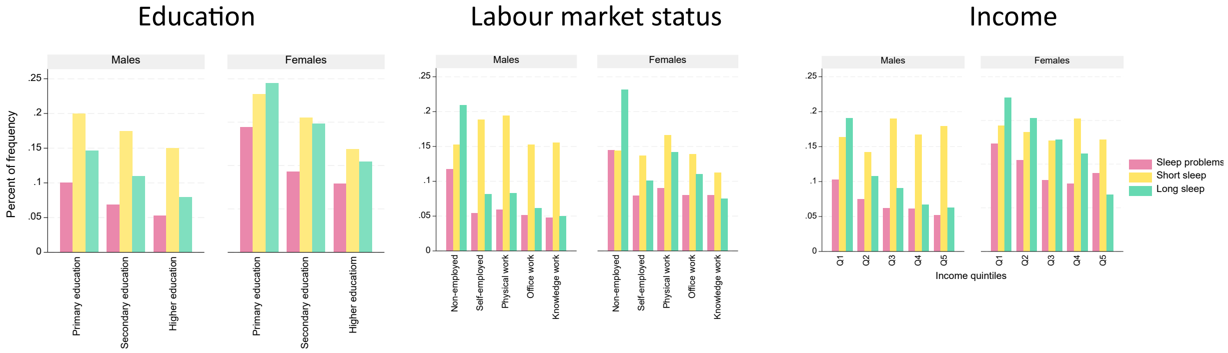

Note: Short and long sleep measures were available only for the pooled 2007-2017 cohorts (N = 8,148)

Table S1 Pairwise correlation coefficients and their statistical significance

	IPGI	SSPGI	LSPGI	SDPGI	Sleep problems	Short sleep	Long sleep	Sleep hours	Education	Labour market status: Non-employed	Labour market status: Self-employed	Labour market status: Physical work	Labour market status: Office work	Labour market status: Knowledge work	Income quintiles	Gender	Age	Birth cohort	Year	PC1	PC2
SSPGI	0.378																				
	0.000																				
LSPGI	0.015	-0.025																			
	0.037	0.001																			
SDPGI	-0.306	-0.726	0.486																		
	0.000	0.000	0.000																		
Sleep problems	0.047	0.045	0.008	-0.030																	
	0.000	0.000	0.257	0.000																	
Short sleep	0.048	0.084	-0.040	-0.099	0.216																
	0.000	0.000	0.000	0.000	0.000																
Long sleep	-0.015	-0.034	0.059	0.078	-0.034	-0.154															
	0.189	0.002	0.000	0.000	0.003	0.000															
Sleep hours	-0.034	-0.090	0.058	0.123	-0.162	-0.617	0.643														
	0.003	0.000	0.000	0.000	0.000	0.000	0.000														
Education	-0.043	-0.038	-0.041	0.006	-0.066	-0.058	-0.072	0.000													
	0.000	0.000	0.000	0.413	0.000	0.000	0.000	0.973													
Labour market status:	0.009	0.004	0.014	0.008	0.095	-0.005	0.167	0.111	-0.210												
Non-employed	0.221	0.568	0.058	0.258	0.000	0.648	0.000	0.000	0.000												
Labour market status:	-0.002	-0.007	-0.010	-0.001	-0.023	0.014	-0.027	-0.028	-0.060	-0.179											
Self-employed	0.771	0.326	0.170	0.932	0.001	0.200	0.018	0.013	0.000	0.000											
Labour market status:	0.017	0.020	0.025	0.001	-0.025	0.044	-0.019	-0.038	-0.270	-0.295	-0.162										
Physical work	0.015	0.005	0.000	0.911	0.001	0.000	0.099	0.001	0.000	0.000	0.000										
Labour market status:	0.004	0.006	-0.002	-0.006	-0.027	-0.016	-0.039	-0.011	0.144	-0.356	-0.196	-0.323									
Office work	0.550	0.402	0.814	0.397	0.000	0.143	0.001	0.308	0.000	0.000	0.000	0.000									
Labour market status:	-0.032	-0.028	-0.033	-0.002	-0.033	-0.029	-0.086	-0.042	0.404	-0.261	-0.144	-0.237	-0.286								
Knowledge work	0.000	0.000	0.000	0.736	0.000	0.010	0.000	0.000	0.000	0.000	0.000	0.000	0.000								
Income	-0.029	-0.026	-0.025	0.002	-0.056	0.009	-0.126	-0.079	0.334	-0.412	-0.028	-0.051	0.146	0.368							
quintiles	0.000	0.000	0.000	0.838	0.000	0.405	0.000	0.000	0.000	0.000	0.000	0.000	0.000	0.000							
Gender	0.002	0.011	0.021	0.001	0.047	-0.041	0.041	0.066	0.083	-0.036	-0.085	-0.146	0.231	-0.011	-0.031						
	0.763	0.114	0.003	0.868	0.000	0.000	0.000	0.000	0.000	0.000	0.000	0.000	0.000	0.121	0.000						
Age	-0.006	0.002	0.003	-0.003	0.120	0.071	0.002	-0.032	-0.263	0.211	0.029	-0.076	-0.103	-0.059	-0.017	-0.035					
	0.370	0.753	0.713	0.709	0.000	0.000	0.841	0.003	0.000	0.000	0.000	0.000	0.000	0.000	0.018	0.000					
Birth cohort	0.007	-0.002	0.010	0.011	-0.083	-0.033	-0.041	0.001	0.341	-0.198	-0.019	0.035	0.090	0.096	0.022	0.023	-0.795				
	0.307	0.760	0.155	0.121	0.000	0.003	0.000	0.937	0.000	0.000	0.007	0.000	0.000	0.000	0.002	0.001	0.000				
Year	0.004	-0.001	0.017	0.012	0.029	0.115	-0.129	-0.099	0.221	-0.052	0.008	-0.045	0.016	0.083	0.023	-0.011	0.038	0.540			
	0.544	0.893	0.018	0.088	0.000	0.000	0.000	0.000	0.000	0.000	0.284	0.000	0.028	0.000	0.001	0.122	0.000	0.000			
PC1	0.012	0.069	0.002	-0.079	0.023	0.035	-0.019	-0.034	0.056	-0.061	-0.013	-0.014	0.011	0.081	0.087	-0.002	-0.008	0.032	0.046		
	0.082	0.000	0.774	0.000	0.001	0.002	0.090	0.002	0.000	0.000	0.070	0.046	0.124	0.000	0.000	0.835	0.276	0.000	0.000		
PC2	0.015	0.031	0.007	-0.014	0.007	-0.018	0.001	0.006	-0.045	0.000	-0.005	0.013	-0.001	-0.010	-0.017	0.005	0.007	-0.063	-0.102	0.044	
	0.034	0.000	0.356	0.044	0.297	0.096	0.952	0.591	0.000	0.977	0.519	0.072	0.910	0.176	0.016	0.446	0.346	0.000	0.000	0.000	
PC3	0.026	0.026	0.040	0.008	-0.011	-0.003	0.013	0.006	0.010	0.009	-0.010	-0.004	0.007	-0.008	-0.022	0.010	-0.027	0.039	0.033	0.001	0.035
	0.000	0.000	0.000	0.285	0.108	0.781	0.256	0.605	0.173	0.188	0.181	0.626	0.308	0.240	0.002	0.171	0.000	0.000	0.000	0.935	0.000

Note: Polygenic indices (PGI) for insomnia (IPGI) (26), short sleep (SSPGI) (10), long sleep (LSPGI) (10), and sleep duration (SDPGI) (10)

Table S2 Detailed descriptive statistics of the pooled FR 1992-2007 and FH 2017 sample

		All		IPGI D1[a]		IPGI D10[a]		SSPGI D1[a]		SSPGI D10[a]		LSPGI D1[a]		LSPGI D10[a]		SDPGI D1[a]		SDPGI D10[a]	
Variable	Explanation	Mean / %	Min<br>Max<br>SD	Mean / %	Min<br>Max<br>SD	Mean / %	Min<br>Max<br>SD	Mean / %	Min<br>Max<br>SD	Mean / %	Min<br>Max<br>SD	Mean / %	Min<br>Max<br>SD	Mean / %	Min<br>Max<br>SD	Mean / %	Min<br>Max<br>SD	Mean / %	Min<br>Max<br>SD
N		20,121		2,045		2,034		1,965		2,016		2,016		2,002		2,048		2,028	
IPGI		-0.001	-3.72	-1.754	-3.72	1.750	1.28	-0.657	-3.64	0.668	-2.46	-0.021	-3.49	0.048	-3.15	0.544	-2.91	-0.536	-3.72
			4.28		-1.29		4.28		2.40		4.28		3.25		3.28		4.28		2.53
			1.002		0.414		0.403		0.946		0.943		1.013		1.012		0.962		0.974
SSPGI		0.001	-4.02	-0.653	-3.36	0.639	-3.09	-1.767	-4.02	1.739	1.27	0.065	-3.34	-0.009	-3.06	1.269	-0.90	-1.249	-4.02
			4.03		2.26		3.78		-1.29		4.03		3.53		3.58		4.03		1.10
			0.998		0.930		0.951		0.407		0.414		1.001		1.001		0.736		0.755
LSPGI		0.002	-3.96	-0.046	-3.14	0.012	-3.54	0.040	-3.48	-0.641	-3.31	-1.756	-3.96	1.758	1.28	-0.834	-3.96	0.846	-2.18
			4.09		3.18		3.47		3.79		3.07		-1.29		4.09		2.23		4.09
			0.999		0.994		1.020		0.998		1.003		0.397		0.413		0.893		0.898
SDPGI		-0.003	-3.87	0.526	-2.63	-0.534	-3.62	1.275	-1.10	-1.301	-3.87	-0.870	-3.80	0.832	-1.97	-1.768	-3.87	1.741	1.28
			4.32		4.32		3.36		4.32		0.89		2.06		4.32		-1.29		4.32
			1.004		0.973		0.971		0.750		0.747		0.901		0.897		0.418		0.409
Sleep hours[b]		7.530	0.00	7.619	0.00	7.424	0.00	7.723	0.00	7.270	0.00	7.435	0.00	7.691	0.00	7.279	0.00	7.778	0.00
			16.00		15.00		14.00		16.00		13.50		16.00		12.75		13.50		15.00
			1.206		1.151		1.236		1.241		1.369		1.162		1.263		1.115		1.263
Short sleep (Yes=1)[b]		15%		12%		20%		10%		22%		17%		13%		21%		9%	
Long sleep (Yes=1)[b]		12%		13%		11%		15%		10%		8%		17%		8%		17%	
Sleep problems (Often=1)[c]		8%		7%		11%		6%		11%		9%		10%		10%		8%	
Education																			
	1 Primary (reference)	20%		17%		21%		17%		22%		19%		22%		20%		20%	
	2 Secondary	42%		40%		45%		41%		42%		40%		45%		43%		43%	
	3 Higher	38%		44%		34%		42%		35%		41%		34%		37%		37%	
Labour market status[d]																			
	1 Non-employed	25%		24%		25%		24%		25%		25%		26%		23%		26%	
	2 Self-employed	9%		8%		9%		9%		9%		9%		9%		10%		9%	
	3 Physical work	21%		19%		22%		19%		23%		19%		23%		22%		21%	
	4 Office work	28%		27%		27%		29%		28%		28%		28%		29%		28%	
	5 Knowledge work	17%		21%		17%		20%		15%		18%		14%		17%		17%	
Income quintile																			
	1 Lowest	22%[e]		20%		24%		20%		23%		20%		24%		21%		22%	
	2 Lower	20%		18%		20%		18%		20%		21%		21%		22%		20%	
	3 Medium	19%[e]		20%		19%		20%		19%		21%		19%		19%		21%	
	4 Higher	20%		21%		20%		21%		19%		18%		19%		20%		19%	
	5 Highest	20%		22%		18%		21%		19%		19%		18%		19%		18%	
Gender	Male=0 (reference)	46%		46%		46%		45%		45%		47%		44%		46%		45%	
Age		44.9	25	44.9	25	44.5	25	44.6	25	44.9	25	44.5	25	45.2	25	45.0	25	44.9	25
			64		64		64		64		64		64		64		64		64
			11.7		11.7		11.5		11.6		11.4		11.8		11.6		11.545		11.756

[a]*All sample members in the 1st decile (D1) and 10th decile (D10) of each polygenic index (PGI): insomnia (IPGI) (26), short sleep (SSPGI) (10), long sleep (LSPGI) (10), and sleep duration (SDPGI) (10)*

[b] *Sleep hours were available only for FR 2007 and 2012, and FH 2017 cohorts (N = 8,148). These values were self-reported and thus subject to misreporting biases, including 0 values. However, outliers were not ample, as illustrated in the histogram in SM Figure 4. The self-reported sleep hours were used to construct binary indicators for short sleep (Yes=1 if <7 hours) and long sleep (Yes=1 if ≥9 hours).*

[c] *Sleep problems were assessed using responses to the survey question: "Do you have trouble sleeping?" (FR) or "Over the past month, how often have you had trouble sleeping?" (FH). Responses were harmonised and dichotomised into two categories: "Often" (indicating sleep problems) and a combined reference category of "Sometimes" and "Not at all".*

[d]*"Labour market status" encompasses both employment status (designated as category 1 "Non-employed") and occupational groups (categories 2-5). Specifically, category 2, "Self-employed," includes individuals classified as "Self-employed persons"; category 3, "Physical work," comprises those classified as "Manual workers"; category 4, "Office work," includes individuals classified as "Lower-level employees with administrative and clerical occupations"; and category 5, "Knowledge work," encompasses those classified as "Upper-level employees with administrative, managerial, professional, and related occupations" according to the Statistics Finland registry data.*

[e]*Some income quintile sizes in the full sample differ slightly from 20% because income was measured in range categories in the FR and FH surveys, which does not allow for allocation of participants into income groups of exactly 20%.*

Table S3 Coefficient estimates from ordered probit regression models of education by gender in the pooled 1992-2017 sample

**Sample**	**Males**	**Females**	**Males**	**Females**	**Males**	**Females**	**Males**	**Females**
**PGI**	**IPGI**	**IPGI**	**SSPGI**	**SSPGI**	**LSPGI**	**LSPGI**	**SDPGI**	**SDPGI**
Model	oprobit	oprobit	oprobit	oprobit	oprobit	oprobit	oprobit	oprobit
N	9,248	10,873	9,248	10,873	9,248	10,873	9,248	10,873
**Dependent variable: Education**								
Explanatory variables:								
PGI	-0.055***	-0.058***	-0.050***	-0.057***	-0.057***	-0.055***	0.005	0.012
	(0.012)	(0.011)	(0.012)	(0.011)	(0.012)	(0.011)	(0.012)	(0.011)
PC1	11.656***	6.979***	12.166***	7.299***	11.581***	6.901***	11.688***	6.917***
	(1.648)	(1.587)	(1.654)	(1.590)	(1.650)	(1.587)	(1.660)	(1.593)
PC2	-2.645	-5.560***	-2.489	-5.515***	-2.606	-5.715***	-2.688	-5.710***
	(1.624)	(1.655)	(1.619)	(1.657)	(1.617)	(1.659)	(1.623)	(1.659)
PC3	-0.388	0.679	-0.362	0.630	-0.330	0.843	-0.543	0.441
	(1.570)	(1.544)	(1.568)	(1.541)	(1.562)	(1.544)	(1.565)	(1.543)
Birth cohort dummies	Yes	Yes	Yes	Yes	Yes	Yes	Yes	Yes
Cut: primary/secondary education	-0.841***	-1.009***	-0.842***	-1.011***	-0.842***	-1.010***	-0.842***	-1.008***
	(0.024)	(0.025)	(0.024)	(0.025)	(0.024)	(0.024)	(0.024)	(0.025)
Cut: secondary/higher education	0.445***	0.257***	0.443***	0.254***	0.444***	0.255***	0.442***	0.255***
	(0.024)	(0.023)	(0.024)	(0.023)	(0.024)	(0.023)	(0.024)	(0.023)
Pseudo-R^2	0.045	0.092	0.045	0.092	0.045	0.092	0.044	0.091
$p(\chi^2)$	***	***	***	***	***	***	***	***

** P < 0.05, ** P < 0.005, *** P < 0.001; robust standard errors (SE) in parentheses; polygenic indices (PGI) for insomnia (IPGI) (26), short sleep (SSPGI) (10), long sleep (LSPGI) (10), and sleep duration (SDPGI) (10)*

Table S4 Coefficient estimates from ordered probit regression models of education in the pooled 1992-2017 sample

**Sample**	**All**	**All**	**All**	**All**	**All**	**All**	**All**	**All**
**PGI**	**IPGI**	**IPGI**	**SSPGI**	**SSPGI**	**LSPGI**	**LSPGI**	**SDPGI**	**SDPGI**
**PGI specification**	**Linear + squared**	**Linear + birth cohort interactions**	**Linear + squared**	**Linear + birth cohort interactions**	**Linear + squared**	**Linear + birth cohort interactions**	**Linear + squared**	**Linear + birth cohort interactions**
Model	oprobit	oprobit	oprobit	oprobit	oprobit	oprobit	oprobit	oprobit
N	20,121	20,121	20,121	20,121	20,121	20,121	20,121	20,121
**Dependent variable: Education**								
Explanatory variables:								
PGI	-0.057***	-0.061***	-0.054***	-0.046**	-0.057***	-0.057***	0.008	-0.004
	(0.008)	(0.016)	(0.008)	(0.015)	(0.008)	(0.016)	(0.008)	(0.016)
PGI squared	0.013*		0.002		0.008		0.001	
	(0.006)		(0.006)		(0.006)		(0.006)	
Female	0.196***	0.196***	0.197***	0.197***	0.198***	0.198***	0.195***	0.195***
	(0.016)	(0.016)	(0.016)	(0.016)	(0.016)	(0.016)	(0.016)	(0.016)
Birth cohort 1920-1930ies	-0.976***	-0.976***	-0.979***	-0.979***	-0.979***	-0.978***	-0.976***	-0.977***
	(0.035)	(0.035)	(0.035)	(0.035)	(0.035)	(0.035)	(0.035)	(0.035)
Birth cohort 1940ies	-0.439***	-0.439***	-0.440***	-0.440***	-0.440***	-0.440***	-0.440***	-0.440***
	(0.027)	(0.027)	(0.027)	(0.027)	(0.027)	(0.027)	(0.027)	(0.027)
Birth cohort 1950ies	Reference	Reference	Reference	Reference	Reference	Reference	Reference	Reference
Birth cohort 1960ies	0.265***	0.266***	0.263***	0.263***	0.263***	0.263***	0.263***	0.263***
	(0.022)	(0.022)	(0.022)	(0.022)	(0.022)	(0.022)	(0.022)	(0.022)
Birth cohort 1970ies	0.402***	0.401***	0.399***	0.399***	0.400***	0.400***	0.398***	0.398***
	(0.024)	(0.024)	(0.024)	(0.024)	(0.024)	(0.024)	(0.024)	(0.024)
Birth cohort 1980-1990ies	0.377***	0.376***	0.373***	0.373***	0.376***	0.376***	0.375***	0.375***
	(0.030)	(0.030)	(0.030)	(0.030)	(0.029)	(0.030)	(0.030)	(0.030)
PGI # Birth cohort 1920-1930ies		0.012		0.002		-0.003		-0.043
		(0.035)		(0.036)		(0.034)		(0.035)
PGI # Birth cohort 1940ies		-0.014		0.012		-0.010		0.003
		(0.026)		(0.026)		(0.027)		(0.026)
PGI # Birth cohort 1960ies		-0.009		-0.037		0.002		0.048*
		(0.021)		(0.022)		(0.022)		(0.022)
PGI # Birth cohort 1970ies		0.045		-0.006		0.024		0.024
		(0.024)		(0.024)		(0.024)		(0.025)
PGI # Birth cohort 1980-1990ies		0.017		-0.005		-0.020		0.013
		(0.031)		(0.030)		(0.030)		(0.029)
PC1	9.214***	9.188***	9.612***	9.662***	9.113***	9.127***	9.189***	9.218***
	(1.142)	(1.143)	(1.145)	(1.145)	(1.142)	(1.142)	(1.148)	(1.148)
PC2	-4.086***	-4.111***	-3.986***	-3.987***	-4.153***	-4.121***	-4.179***	-4.168***
	(1.162)	(1.164)	(1.163)	(1.162)	(1.163)	(1.164)	(1.164)	(1.164)
PC3	0.139	0.127	0.108	0.095	0.222	0.222	-0.077	-0.106
	(1.099)	(1.100)	(1.097)	(1.098)	(1.097)	(1.097)	(1.097)	(1.098)
Cut: Primary/Secondary	-0.811***	-0.824***	-0.822***	-0.825***	-0.817***	-0.824***	-0.823***	-0.824***
	(0.020)	(0.019)	(0.020)	(0.019)	(0.020)	(0.019)	(0.020)	(0.019)
Cut: Secondary/Higher	0.460***	0.447***	0.448***	0.446***	0.454***	0.447***	0.446***	0.445***
	(0.019)	(0.018)	(0.019)	(0.018)	(0.019)	(0.018)	(0.019)	(0.018)
Pseudo-R^2	0.071	0.071	0.071	0.071	0.071	0.071	0.070	0.070
p(χ^2)	***	***	***	***	***	***	***	***

** P < 0.05, ** P < 0.005, *** P < 0.001; robust SE in parentheses; polygenic indices (PGI) for insomnia (IPGI) (26), short sleep (SSPGI) (10), long sleep (LSPGI) (10), and sleep duration (SDPGI) (10)*

Table S5 Coefficient estimates from ordered probit regression models of education in the pooled 1992-2017 sample

**Sample**	**All**	**Males**	**Females**	**All**	**Males**	**Females**	**All**	**Males**	**Females**
**PGI**	**IPGI**	**IPGI**	**IPGI**	**SSPGI**	**SSPGI**	**SSPGI**	**LSPGI**	**LSPGI**	**LSPGI**
Model	oprobit	oprobit	oprobit	oprobit	oprobit	oprobit	oprobit	oprobit	oprobit
**Dependent variable: Education**									
Explanatory variables:									
PGI Decile 1	Reference	Reference	Reference	Reference	Reference	Reference	Reference	Reference	Reference
PGI Decile 2	-0.146***	-0.066	-0.219***	-0.088*	-0.030	-0.142**	0.002	0.027	-0.013
	(0.036)	(0.052)	(0.051)	(0.036)	(0.053)	(0.050)	(0.037)	(0.054)	(0.050)
PGI Decile 3	-0.145***	-0.072	-0.210***	-0.081*	-0.124*	-0.041	-0.047	-0.018	-0.076
	(0.036)	(0.053)	(0.050)	(0.036)	(0.053)	(0.050)	(0.036)	(0.053)	(0.050)
PGI Decile 4	-0.127***	-0.131*	-0.126*	-0.140***	-0.144*	-0.131*	-0.068	-0.080	-0.058
	(0.036)	(0.053)	(0.050)	(0.036)	(0.053)	(0.050)	(0.037)	(0.054)	(0.050)
PGI Decile 5	-0.178***	-0.151**	-0.206***	-0.124***	-0.112*	-0.136*	-0.078*	-0.092	-0.056
	(0.037)	(0.053)	(0.050)	(0.037)	(0.054)	(0.051)	(0.037)	(0.054)	(0.050)
PGI Decile 6	-0.174***	-0.087	-0.254***	-0.126***	-0.101	-0.150**	-0.077*	-0.061	-0.086
	(0.036)	(0.053)	(0.050)	(0.036)	(0.053)	(0.050)	(0.036)	(0.053)	(0.050)
PGI Decile 7	-0.221***	-0.179***	-0.252***	-0.110**	-0.105*	-0.103*	-0.134***	-0.129*	-0.130*
	(0.036)	(0.052)	(0.050)	(0.036)	(0.053)	(0.050)	(0.036)	(0.052)	(0.050)
PGI Decile 8	-0.186***	-0.115*	-0.249***	-0.124***	-0.081	-0.154**	-0.125***	-0.110*	-0.129*
	(0.036)	(0.051)	(0.051)	(0.036)	(0.054)	(0.049)	(0.036)	(0.054)	(0.049)
PGI Decile 9	-0.201***	-0.186***	-0.220***	-0.190***	-0.156**	-0.222***	-0.136***	-0.121*	-0.144**
	(0.036)	(0.053)	(0.050)	(0.036)	(0.054)	(0.049)	(0.036)	(0.053)	(0.049)
PGI Decile 10	-0.245***	-0.195***	-0.289***	-0.206***	-0.183***	-0.230***	-0.179***	-0.163**	-0.188***
	(0.036)	(0.052)	(0.049)	(0.036)	(0.054)	(0.050)	(0.036)	(0.054)	(0.049)
Female	0.196***			0.196***			0.198***		
	(0.016)			(0.016)			(0.016)		
PC1	9.228***	11.652***	7.010***	9.513***	12.044***	7.192***	9.142***	11.637***	6.874***
	(1.142)	(1.650)	(1.588)	(1.145)	(1.652)	(1.591)	(1.143)	(1.651)	(1.589)
PC2	-4.095***	-2.632	-5.511***	-4.037***	-2.547	-5.576***	-4.143***	-2.575	-5.744***
	(1.164)	(1.622)	(1.659)	(1.163)	(1.623)	(1.656)	(1.164)	(1.620)	(1.660)
PC3	0.152	-0.386	0.782	0.121	-0.396	0.684	0.218	-0.312	0.798
	(1.099)	(1.572)	(1.544)	(1.098)	(1.570)	(1.542)	(1.097)	(1.565)	(1.545)
Birth cohort dummies	Yes	Yes	Yes	Yes	Yes	Yes	Yes	Yes	Yes
Cut: Primary/Secondary	-0.987***	-0.960***	-1.212***	-0.944***	-0.946***	-1.142***	-0.909***	-0.917***	-1.098***
	(0.031)	(0.043)	(0.042)	(0.031)	(0.044)	(0.042)	(0.031)	(0.044)	(0.041)
Cut: Secondary/Higher	0.284***	0.326***	0.056	0.326***	0.340***	0.124**	0.362***	0.369***	0.167***
	(0.030)	(0.042)	(0.041)	(0.031)	(0.043)	(0.041)	(0.031)	(0.044)	(0.040)
Pseudo-R^2	0.072	0.046	0.093	0.071	0.045	0.092	0.071	0.045	0.092
$p(\chi^2)$	***	***	***	***	***	***	***	***	***
N	20,121	9,248	10,873	20,121	9,248	10,873	20,121	9,248	10,873

** P < 0.05, ** P < 0.005, *** P < 0.001; robust SE in parentheses; polygenic indices (PGI) for insomnia (IPGI) (26), short sleep (SSPGI) (10), and long sleep (LSPGI) (10)*

Table S6 Differences in predicted probabilities of having a specific level of education in the IPGI, SSPGI and LSPGI top three deciles compared to the lowest decile in the pooled 1992-2017 sample

**Sample**	**All**	**Males**	**Females**	**All**	**Males**	**Females**	**All**	**Males**	**Females**
**PGI**	**IPGI**	**IPGI**	**IPGI**	**SSPGI**	**SSPGI**	**SSPGI**	**LSPGI**	**LSPGI**	**LSPGI**
Total N in the model	20,121	9,248	10,873	20,121	9,248	10,873	20,121	9,248	10,873
**Reference: 1st decile PGI**									
Average marginal effects:									
**8th decile PGI**									
Primary education	0.044***	0.030*	0.054***	0.030***	0.022	0.034**	0.031***	0.030*	0.029*
	(0.009)	(0.014)	(0.011)	(0.009)	(0.014)	(0.011)	(0.009)	(0.015)	(0.011)
Secondary education	0.021***	0.010*	0.034***	0.014***	0.007	0.020**	0.013***	0.008*	0.016*
	(0.004)	(0.005)	(0.007)	(0.004)	(0.005)	(0.007)	(0.004)	(0.004)	(0.006)
Higher education	-0.066***	-0.040*	-0.087***	-0.044***	-0.028	-0.054**	-0.044***	-0.038*	-0.045*
	(0.013)	(0.018)	(0.018)	(0.013)	(0.019)	(0.017)	(0.013)	(0.019)	(0.017)
**9th decile PGI**									
Primary education	0.048***	0.051***	0.047***	0.047***	0.043**	0.050***	0.034***	0.033*	0.033**
	(0.009)	(0.015)	(0.011)	(0.009)	(0.015)	(0.011)	(0.009)	(0.015)	(0.011)
Secondary education	0.023***	0.013**	0.031***	0.019***	0.011*	0.027***	0.014***	0.008*	0.018**
	(0.004)	(0.004)	(0.007)	(0.004)	(0.004)	(0.006)	(0.004)	(0.004)	(0.006)
Higher education	-0.071***	-0.064***	-0.078***	-0.066***	-0.054**	-0.078***	-0.047***	-0.042*	-0.050**
	(0.013)	(0.018)	(0.018)	(0.013)	(0.019)	(0.017)	(0.013)	(0.018)	(0.017)
**10th decile PGI**									
Primary education	0.060***	0.053***	0.063***	0.051***	0.050***	0.052***	0.045***	0.046**	0.043***
	(0.009)	(0.014)	(0.011)	(0.009)	(0.015)	(0.011)	(0.009)	(0.015)	(0.011)
Secondary education	0.026***	0.014***	0.038***	0.021***	0.012**	0.028***	0.017***	0.010*	0.022***
	(0.004)	(0.004)	(0.007)	(0.004)	(0.004)	(0.006)	(0.004)	(0.004)	(0.006)
Higher education	-0.086***	-0.067***	-0.101***	-0.072***	-0.063***	-0.080***	-0.062***	-0.056**	-0.066***
	(0.012)	(0.018)	(0.017)	(0.013)	(0.019)	(0.017)	(0.013)	(0.018)	(0.017)

** P < 0.05, ** P < 0.005, *** P < 0.001; robust SE in parentheses; average marginal effects from the models in SM Table 7; polygenic indices (PGI) for insomnia (IPGI) (26), short sleep (SSPGI) (10), and long sleep (LSPGI) (10)*

Table S7 Coefficient estimates from extended regression models of educational attainment, belonging to the knowledge work occupational group, and income quintiles

**Sample**	**All**	**All**	**All**	**All**	**All**	**All**	**All**	**All**	**All**
**Phenotypic sleep trait**	**Sleep problems**	**Short sleep**	**Long sleep**	**Sleep problems**	**Short sleep**	**Long sleep**	**Sleep problems**	**Short sleep**	**Long sleep**
**PGI as instrument for the phenotypic sleep trait**	**IPGI**	**SSPGI**	**LSPGI**	**IPGI**	**SSPGI**	**LSPGI**	**IPGI**	**SSPGI**	**LSPGI**
Model	ERM	ERM	ERM	ERM	ERM	ERM	ERM	ERM	ERM
Estimation	eoprobit	eoprobit	eoprobit	eprobit	eprobit	eprobit	eoprobit	eoprobit	eoprobit
N	19,965	8,148	8,148	19,554	7,971	7,971	19,555	7,969	7,969
**Main equation dependent variable:**	**Education**	**Education**	**Education**	**Knowledge work**	**Knowledge work**	**Knowledge work**	**Income**	**Income**	**Income**
Explanatory variables:									
Phenotypic sleep trait (endogenous)	-0.273***	-0.687***	-0.695***	-1.287***	-0.961***	-1.594***	-0.402***	-0.413*	-0.051
	(0.078)	(0.125)	(0.210)	(0.368)	(0.272)	(0.227)	(0.100)	(0.153)	(0.330)
Female	0.205***	0.297***	0.336***	-0.008	-0.020	0.047	-0.065***	-0.083**	-0.102***
	(0.017)	(0.028)	(0.027)	(0.029)	(0.035)	(0.034)	(0.016)	(0.026)	(0.026)
Phenotypic sleep trait (endogenous) # Female	0.009	-0.047	-0.065	0.177*	-0.077	0.075	0.146*	-0.065	0.175*
	(0.062)	(0.069)	(0.079)	(0.076)	(0.083)	(0.095)	(0.059)	(0.066)	(0.079)
Age	0.008	0.070***	0.045**	0.122***	0.158***	0.091**	0.168***	0.208***	0.205***
	(0.007)	(0.014)	(0.015)	(0.011)	(0.018)	(0.028)	(0.007)	(0.013)	(0.014)
Age squared	-0.000***	-0.001***	-0.001***	-0.001***	-0.002***	-0.001***	-0.002***	-0.002***	-0.002***
	(0.000)	(0.000)	(0.000)	(0.000)	(0.000)	(0.000)	(0.000)	(0.000)	(0.000)
PC1	9.096***	4.519**	3.724*	16.939***	9.509***	7.344***	14.194***	6.013***	5.611***
	(1.159)	(1.567)	(1.550)	(1.490)	(1.816)	(1.915)	(1.111)	(1.470)	(1.462)
PC2	-3.536**	-5.405***	-5.184***	-1.168	-1.508	-1.158	-2.893*	-5.644***	-5.411***
	(1.196)	(1.612)	(1.572)	(1.452)	(1.672)	(1.669)	(1.247)	(1.477)	(1.487)
PC3	-0.318	2.245	2.869	-2.765*	0.128	1.691	-3.094**	0.211	0.287
	(1.118)	(1.469)	(1.471)	(1.358)	(1.666)	(1.657)	(1.094)	(1.382)	(1.390)
Year dummies	Yes	Yes	Yes	Yes	Yes	Yes	Yes	Yes	Yes
Birth cohort dummies	Yes	Yes	Yes	Yes	Yes	Yes	Yes	Yes	Yes
Constant				-3.424***	-3.903***	-2.392***			
				(0.273)	(0.457)	(0.661)			
**Endogenous explanatory variable: Phenotypic sleep trait**									
Estimation	probit	probit	probit	probit	probit	probit	probit	probit	probit
Explanatory variables:									
PGI	0.050*	0.141***	0.128***	0.057*	0.131***	0.131***	0.052*	0.139***	0.104**
	(0.021)	(0.026)	(0.030)	(0.021)	(0.026)	(0.028)	(0.022)	(0.026)	(0.034)
Female	0.180***	-0.123***	0.146***	0.183***	-0.123***	0.153***	0.175***	-0.123***	0.144***
	(0.027)	(0.035)	(0.038)	(0.027)	(0.035)	(0.038)	(0.027)	(0.035)	(0.038)
PGI # Female	0.066*	-0.005	-0.038	0.059*	-0.006	-0.047	0.067*	0.002	-0.020
	(0.027)	(0.035)	(0.038)	(0.027)	(0.034)	(0.037)	(0.027)	(0.035)	(0.043)
Age	0.037*	0.079***	-0.120***	0.038**	0.078***	-0.116***	0.038**	0.076***	-0.124***
	(0.013)	(0.021)	(0.022)	(0.013)	(0.021)	(0.023)	(0.013)	(0.021)	(0.022)
Age squared	-0.000*	-0.001***	0.001***	-0.000*	-0.001***	0.001***	-0.000*	-0.001***	0.001***
	(0.000)	(0.000)	(0.000)	(0.000)	(0.000)	(0.000)	(0.000)	(0.000)	(0.000)
PC1	5.453**	3.067	-1.738	6.032***	4.065*	-3.142	5.371**	3.303	-0.482
	(1.774)	(1.983)	(2.735)	(1.801)	(1.996)	(2.690)	(1.795)	(1.998)	(2.509)
PC2	1.493	-2.874	-1.433	0.907	-3.258	-2.778	1.201	-2.479	-1.525
	(1.732)	(1.873)	(2.459)	(1.831)	(1.908)	(2.610)	(1.791)	(1.931)	(2.318)
PC3	-2.738	-0.832	4.908*	-2.340	-0.828	5.661*	-3.154	-0.915	3.864
	(1.746)	(1.828)	(2.290)	(1.803)	(1.845)	(2.398)	(1.776)	(1.856)	(2.328)
Birth cohort dummies	Yes	Yes	Yes	Yes	Yes	Yes	Yes	Yes	Yes
Year dummies	Yes	Yes	Yes	Yes	Yes	Yes	Yes	Yes	Yes
Correlation of errors: Main equation / phenotypic sleep trait	0.035	0.298***	0.270*	0.568*	0.520**	0.719***	0.050	0.222*	-0.207
	(0.027)	(0.064)	(0.105)	(0.232)	(0.173)	(0.174)	(0.042)	(0.082)	(0.166)
Log-pseudolikelihood	-25027	-10768	-10196	-14149	-7327	-6737	-36125	-15784	-15193
$p(\chi^2)$	***	***	***	***	***	***	***	***	***

** P < 0.05, ** P < 0.005, *** P < 0.001; robust SE in parentheses; polygenic indices (PGI) for insomnia (IPGI) (26), short sleep (SSPGI) (10), and long sleep (LSPGI) (10); short and long sleep variables were available only for FR 2007 and 2012, and FH 2017 cohorts*

Table S8 Coefficient estimates from extended regression models of educational attainment, belonging to the knowledge work occupational group, and income quintiles in the pooled 2012-2017 sample: Sleep Duration Polygenic Index (SDPGI)

**Sample**	**All**	**All**	**All**
**Phenotypic sleep trait**	**Sleep hours**	**Sleep hours**	**Sleep hours**
**PGI as instrument for the phenotypic sleep trait**	**SDPGI**	**SDPGI**	**SDPGI**
Model	ERM	ERM	ERM
Estimation	eoprobit	eprobit	eoprobit
N			
**Main equation dependent variable:**	**Education**	**Knowledge work**	**Income quintiles**
Explanatory variables:			
Phenotypic sleep hours (endogenous)	0.212*	-0.029	0.129
	(0.082)	(0.110)	(0.078)
Female	0.270***	0.008	-0.099***
	(0.033)	(0.037)	(0.026)
Age	0.075***	0.150***	0.210***
	(0.015)	(0.021)	(0.014)
Age squared	-0.001***	-0.002***	-0.002***
	(0.000)	(0.000)	(0.000)
PC1	4.413**	8.572***	5.865***
	(1.555)	(1.913)	(1.465)
PC2	-4.910**	-0.705	-5.387***
	(1.562)	(1.718)	(1.462)
PC3	2.076	0.312	0.122
	(1.448)	(1.717)	(1.372)
Year dummies	Yes	Yes	Yes
Birth cohort dummies	Yes	Yes	Yes
Cut (dependent variable categories 1/2)	1.905*		4.688***
	(0.825)		(0.716)
Cut (dependent variable categories 2/3)	3.332***		5.314***
	(0.792)		(0.706)
Cut (dependent variable categories 3/4)			5.773***
			(0.698)
Cut (dependent variable categories 4/5)			6.389***
			(0.688)
Constant		-3.630***	
		(1.098)	
**Endogenous explanatory variable: Phenotypic sleep hours**			
Estimation	Linear regression	Linear regression	Linear regression
Explanatory variables:			
SDPGI	0.146***	0.146***	0.148***
	(0.013)	(0.013)	(0.013)
Female	0.157***	0.164***	0.154***
	(0.026)	(0.027)	(0.026)
Age	-0.075***	-0.076***	-0.072***
	(0.014)	(0.014)	(0.014)
Age squared	0.001***	0.001***	0.001***
	(0.000)	(0.000)	(0.000)
PC1	-1.504	-1.961	-1.537
	(1.375)	(1.390)	(1.392)
PC2	0.215	0.485	0.211
	(1.272)	(1.265)	(1.295)
PC3	0.937	0.708	0.945
	(1.287)	(1.297)	(1.293)
Birth cohort dummies	Yes	Yes	Yes
Year dummies	Yes	Yes	Yes
Constant	9.033***	9.075***	8.948***
	(0.350)	(0.351)	(0.353)
Correlation of errors: main equation outcome / phenotypic sleep trait	-0.255*	-0.020	-0.205*
	(0.097)	(0.130)	(0.091)
Log-pseudolikelihood	-20389	-16694	-25179
p(χ^2)	***	***	***

** P < 0.05, ** P < 0.005, *** P < 0.001; robust SE in parentheses; SDPGI = polygenic index for sleep duration (10)*

Table S9 Coefficient estimates from probit models of belonging to the knowledge work occupational group by genders in the pooled 1992-2017 sample

**Sample**	**Males**	**Females**	**Males**	**Females**	**Males**	**Females**
**PGI**	**IPGI**	**IPGI**	**SSPGI**	**SSPGI**	**LSPGI**	**LSPGI**
Model	probit	probit	probit	probit	probit	probit
N	9,068	10,639	9,068	10,639	9,068	10,639
**Dependent variable: Belonging to the knowledge work occupational category**						
Explanatory variables:						
PGI	-0.044*	-0.059***	-0.047**	-0.060***	-0.086***	-0.025
	(0.016)	(0.015)	(0.016)	(0.015)	(0.016)	(0.014)
Age	0.125***	0.133***	0.125***	0.134***	0.125***	0.133***
	(0.015)	(0.014)	(0.015)	(0.014)	(0.015)	(0.014)
Age squared	-0.001***	-0.002***	-0.001***	-0.002***	-0.001***	-0.002***
	(0.000)	(0.000)	(0.000)	(0.000)	(0.000)	(0.000)
PC1	19.040***	14.356***	19.539***	14.636***	18.997***	14.217***
	(2.146)	(2.026)	(2.156)	(2.034)	(2.149)	(2.024)
PC2	0.196	-3.304	0.287	-3.214	0.222	-3.458
	(2.059)	(2.020)	(2.058)	(2.017)	(2.051)	(2.022)
PC3	-1.158	-3.536	-1.065	-3.630	-0.947	-3.605
	(2.027)	(1.916)	(2.024)	(1.915)	(2.023)	(1.917)
Year dummies	Yes	Yes	Yes	Yes	Yes	Yes
Birth cohort dummies	Yes	Yes	Yes	Yes	Yes	Yes
Constant	-3.608***	-3.601***	-3.613***	-3.603***	-3.621***	-3.607***
	(0.366)	(0.343)	(0.366)	(0.343)	(0.365)	(0.343)
Pseudo-R^2	0.033	0.041	0.033	0.041	0.036	0.039
$p(\chi^2)$	***	***	***	***	***	***

** P < 0.05, ** P < 0.005, *** P < 0.001; robust SE in parentheses; polygenic indices (PGI) for insomnia (IPGI) (26), short sleep (SSPGI) (10), and long sleep (LSPGI) (10)*

Table S10 Coefficient estimates from probit models of belonging to a specific labour market status in the pooled 1992-2017 sample: Insomnia Polygenic Index (IPGI)

**Sample**	**All**	**All**	**All**	**All**	**All**	**All**	**All**	**All**	**All**	**All**
**PGI**	**IPGI**	**IPGI**	**IPGI**	**IPGI**	**IPGI**	**IPGI**	**IPGI**	**IPGI**	**IPGI**	**IPGI**
**PGI specification**	**Linear**	**Linear + squared**	**Linear**	**Linear + squared**	**Linear**	**Linear + squared**	**Linear**	**Linear + squared**	**Linear**	**Linear + squared**
**Labour market status**	**Non-employed**	**Non-employed**	**Self-employed**	**Self-employed**	**Physical work**	**Physical work**	**Office work**	**Office work**	**Knowledge work**	**Knowledge work**
N	19,707	19,707	19,707	19,707	19,707	19,707	19,707	19,707	19,707	19,707
Model	probit	probit	probit	probit	probit	probit	probit	probit	probit	probit
**Dependent variable: Belonging to the specific labour market status**										
Explanatory variables:										
PGI	0.012	0.021*	0.010	-0.004	0.014	0.026*	0.006	0.004	-0.044**	-0.051***
	(0.015)	(0.010)	(0.017)	(0.013)	(0.014)	(0.010)	(0.016)	(0.010)	(0.016)	(0.011)
PGI squared		0.003		-0.016		-0.003		-0.005		0.012
		(0.007)		(0.009)		(0.007)		(0.007)		(0.007)
Female	-0.106***	-0.106***	-0.297***	-0.296***	-0.425***	-0.425***	0.657***	0.657***	-0.037	-0.037
	(0.021)	(0.021)	(0.026)	(0.026)	(0.021)	(0.021)	(0.020)	(0.020)	(0.022)	(0.021)
PGI # Female	0.016		-0.030		0.025		-0.003		-0.015	
	(0.021)		(0.025)		(0.020)		(0.020)		(0.022)	
Age	-0.256***	-0.256***	0.123***	0.123***	0.048***	0.048***	0.066***	0.066***	0.128***	0.128***
	(0.010)	(0.010)	(0.013)	(0.013)	(0.010)	(0.010)	(0.009)	(0.009)	(0.010)	(0.010)
Age squared	0.003***	0.003***	-0.001***	-0.001***	-0.001***	-0.001***	-0.001***	-0.001***	-0.002***	-0.002***
	(0.000)	(0.000)	(0.000)	(0.000)	(0.000)	(0.000)	(0.000)	(0.000)	(0.000)	(0.000)
PC1	-12.911***	-12.901***	-3.193	-3.209	-2.563	-2.541	2.337	2.330	16.627***	16.626***
	(1.502)	(1.502)	(1.806)	(1.807)	(1.433)	(1.433)	(1.361)	(1.361)	(1.472)	(1.472)
PC2	0.720	0.730	-0.734	-0.740	1.570	1.585	-0.654	-0.654	-1.540	-1.553
	(1.697)	(1.696)	(1.865)	(1.870)	(1.472)	(1.472)	(1.372)	(1.373)	(1.438)	(1.438)
PC3	3.924*	3.932*	-2.124	-2.152	-0.496	-0.503	0.175	0.163	-2.452	-2.439
	(1.481)	(1.481)	(1.804)	(1.806)	(1.452)	(1.452)	(1.316)	(1.316)	(1.387)	(1.387)
Year dummies	Yes	Yes	Yes	Yes	Yes	Yes	Yes	Yes	Yes	Yes
Birth cohort dummies	Yes	Yes	Yes	Yes	Yes	Yes	Yes	Yes	Yes	Yes
Constant	3.694***	3.693***	-3.903***	-3.894***	-1.170***	-1.164***	-1.762***	-1.759***	-3.553***	-3.566***
	(0.240)	(0.240)	(0.312)	(0.312)	(0.238)	(0.238)	(0.226)	(0.227)	(0.250)	(0.250)
Pseudo-R^2	0.128	0.128	0.027	0.027	0.035	0.035	0.067	0.067	0.036	0.036
$p(\chi^2)$	***	***	***	***	***	***	***	***	***	***

** P < 0.05, ** P < 0.005, *** P < 0.001; robust SE in parentheses; IPGI = polygenic index (PGI) for insomnia (26)*

Table S11 Coefficient estimates from probit models of belonging to a specific labour market status in the pooled 1992-2017 sample: Short Sleep Polygenic Index (SSPGI)

**Sample**	**All**	**All**	**All**	**All**	**All**	**All**	**All**	**All**	**All**	**All**
**PGI**	**SSPGI**	**SSPGI**	**SSPGI**	**SSPGI**	**SSPGI**	**SSPGI**	**SSPGI**	**SSPGI**	**SSPGI**	**SSPGI**
**PGI specification**	**Linear**	**Linear + squared**	**Linear**	**Linear + squared**	**Linear**	**Linear + squared**	**Linear**	**Linear + squared**	**Linear**	**Linear + squared**
**Labour market status**	**Non-employed**	**Non-employed**	**Self-employed**	**Self-employed**	**Physical work**	**Physical work**	**Office work**	**Office work**	**Knowledge work**	**Knowledge work**
N	19,707	19,707	19,707	19,707	19,707	19,707	19,707	19,707	19,707	19,707
Model	probit	probit	probit	probit	probit	probit	probit	probit	probit	probit
**Dependent variable: Belonging to the specific labour market status**										
Explanatory variables:										
PGI	0.006	0.021*	-0.018	-0.011	0.037*	0.032**	0.003	0.001	-0.044**	-0.053***
	(0.015)	(0.010)	(0.018)	(0.013)	(0.014)	(0.010)	(0.016)	(0.010)	(0.016)	(0.011)
PGI squared		0.003		-0.001		-0.002		0.000		-0.002
		(0.007)		(0.009)		(0.007)		(0.007)		(0.008)
Female	-0.106***	-0.106***	-0.296***	-0.296***	-0.425***	-0.425***	0.657***	0.657***	-0.036	-0.035
	(0.021)	(0.021)	(0.026)	(0.026)	(0.021)	(0.021)	(0.020)	(0.020)	(0.022)	(0.021)
PGI # Female	0.027		0.014		-0.012		-0.002		-0.016	
	(0.021)		(0.026)		(0.021)		(0.020)		(0.021)	
Age	-0.256***	-0.256***	0.123***	0.123***	0.048***	0.048***	0.066***	0.066***	0.128***	0.128***
	(0.010)	(0.010)	(0.013)	(0.013)	(0.010)	(0.010)	(0.009)	(0.009)	(0.010)	(0.010)
Age squared	0.003***	0.003***	-0.001***	-0.001***	-0.001***	-0.001***	-0.001***	-0.001***	-0.002***	-0.002***
	(0.000)	(0.000)	(0.000)	(0.000)	(0.000)	(0.000)	(0.000)	(0.000)	(0.000)	(0.000)
PC1	-13.035***	-13.070***	-3.084	-3.092	-2.810	-2.794	2.328	2.329	17.000***	17.024***
	(1.505)	(1.506)	(1.809)	(1.808)	(1.436)	(1.437)	(1.364)	(1.364)	(1.478)	(1.478)
PC2	0.695	0.681	-0.679	-0.686	1.513	1.518	-0.652	-0.653	-1.452	-1.452
	(1.700)	(1.700)	(1.869)	(1.867)	(1.474)	(1.474)	(1.373)	(1.373)	(1.436)	(1.437)
PC3	3.933*	3.933*	-2.120	-2.122	-0.527	-0.524	0.184	0.184	-2.457	-2.454
	(1.481)	(1.481)	(1.806)	(1.805)	(1.453)	(1.453)	(1.316)	(1.316)	(1.386)	(1.386)
Year dummies	Yes	Yes	Yes	Yes	Yes	Yes	Yes	Yes	Yes	Yes
Birth cohort dummies	Yes	Yes	Yes	Yes	Yes	Yes	Yes	Yes	Yes	Yes
Constant	3.696***	3.696***	-3.912***	-3.910***	-1.159***	-1.159***	-1.762***	-1.763***	-3.556***	-3.557***
	(0.240)	(0.240)	(0.312)	(0.312)	(0.238)	(0.238)	(0.226)	(0.226)	(0.250)	(0.250)
Pseudo-R^2	0.129	0.128	0.027	0.027	0.035	0.035	0.067	0.067	0.036	0.036
p(χ^2)	***	***	***	***	***	***	***	***	***	***

** P < 0.05, ** P < 0.005, *** P < 0.001; robust SE in parentheses; SSPGI = polygenic index (PGI) for short sleep (10)*

Table S12 Coefficient estimates from probit models of belonging to a specific labour market status in the pooled 1992-2017 sample: Long Sleep Polygenic Index (LSPGI)

**Sample**	**All**	**All**	**All**	**All**	**All**	**All**	**All**	**All**	**All**	**All**
**PGI**	**LSPGI**	**LSPGI**	**LSPGI**	**LSPGI**	**LSPGI**	**LSPGI**	**LSPGI**	**LSPGI**	**LSPGI**	**LSPGI**
**PGI specification**	**Linear**	**Linear + squared**	**Linear**	**Linear + squared**	**Linear**	**Linear + squared**	**Linear**	**Linear + squared**	**Linear**	**Linear + squared**
**Labour market status**	**Non-employed**	**Non-employed**	**Self-employed**	**Self-employed**	**Physical work**	**Physical work**	**Office work**	**Office work**	**Knowledge work**	**Knowledge work**
N	19,707	19,707	19,707	19,707	19,707	19,707	19,707	19,707	19,707	19,707
Model	probit	probit	probit	probit	probit	probit	probit	probit	probit	probit
**Dependent variable: Belonging to the specific labour market status**										
Explanatory variables:										
PGI	0.030	0.024*	-0.026	-0.014	0.051***	0.043***	0.009	-0.009	-0.086***	-0.054***
	(0.015)	(0.010)	(0.018)	(0.013)	(0.014)	(0.010)	(0.016)	(0.010)	(0.016)	(0.011)
PGI squared		0.012		0.008		-0.007		-0.003		-0.010
		(0.007)		(0.009)		(0.007)		(0.007)		(0.008)
Female	-0.106***	-0.107***	-0.296***	-0.296***	-0.426***	-0.426***	0.657***	0.657***	-0.031	-0.033
	(0.021)	(0.021)	(0.026)	(0.026)	(0.021)	(0.021)	(0.020)	(0.020)	(0.022)	(0.021)
PGI # Female	-0.010		0.027		-0.018		-0.028		0.061**	
	(0.021)		(0.026)		(0.020)		(0.020)		(0.021)	
Age	-0.256***	-0.256***	0.123***	0.123***	0.048***	0.048***	0.066***	0.066***	0.128***	0.128***
	(0.010)	(0.010)	(0.013)	(0.013)	(0.010)	(0.010)	(0.009)	(0.009)	(0.010)	(0.010)
Age squared	0.003***	0.003***	-0.001***	-0.001***	-0.001***	-0.001***	-0.001***	-0.001***	-0.002***	-0.002***
	(0.000)	(0.000)	(0.000)	(0.000)	(0.000)	(0.000)	(0.000)	(0.000)	(0.000)	(0.000)
PC1	-12.860***	-12.914***	-3.213	-3.241	-2.500	-2.502	2.363	2.353	16.528***	16.575***
	(1.503)	(1.503)	(1.807)	(1.806)	(1.434)	(1.434)	(1.361)	(1.362)	(1.472)	(1.472)
PC2	0.747	0.733	-0.722	-0.738	1.570	1.580	-0.637	-0.636	-1.615	-1.597
	(1.703)	(1.703)	(1.872)	(1.866)	(1.475)	(1.476)	(1.373)	(1.373)	(1.436)	(1.437)
PC3	3.873*	3.842*	-2.100	-2.095	-0.642	-0.640	0.256	0.238	-2.379	-2.315
	(1.481)	(1.481)	(1.808)	(1.806)	(1.453)	(1.453)	(1.318)	(1.318)	(1.386)	(1.386)
Year dummies	Yes	Yes	Yes	Yes	Yes	Yes	Yes	Yes	Yes	Yes
Birth cohort dummies	Yes	Yes	Yes	Yes	Yes	Yes	Yes	Yes	Yes	Yes
Constant	3.702***	3.690***	-3.908***	-3.918***	-1.161***	-1.152***	-1.767***	-1.762***	-3.566***	-3.553***
	(0.240)	(0.240)	(0.312)	(0.312)	(0.238)	(0.238)	(0.226)	(0.227)	(0.250)	(0.250)
Pseudo-R^2	0.129	0.129	0.027	0.027	0.035	0.035	0.067	0.067	0.037	0.036
p(χ^2)	***	***	***	***	***	***	***	***	***	***

** P < 0.05, ** P < 0.005, *** P < 0.001; robust SE in parentheses; LSPGI = polygenic index (PGI) for long sleep (10)*

Table S13 Coefficient estimates from probit models of belonging to a specific labour market status in the pooled 1992-2017 sample: Sleep Duration Polygenic Index (SDPGI)

**Sample**	**All**	**All**	**All**	**All**	**All**	**All**	**All**	**All**	**All**	**All**
**PGI**	**SDPGI**	**SDPGI**	**SDPGI**	**SDPGI**	**SDPGI**	**SDPGI**	**SDPGI**	**SDPGI**	**SDPGI**	**SDPGI**
**PGI specification**	**Linear**	**Linear + squared**	**Linear**	**Linear + squared**	**Linear**	**Linear + squared**	**Linear**	**Linear + squared**	**Linear**	**Linear + squared**
**Labour market status**	**Non-employed**	**Non-employed**	**Self-employed**	**Self-employed**	**Physical work**	**Physical work**	**Office work**	**Office work**	**Knowledge work**	**Knowledge work**
N	19,707	19,707	19,707	19,707	19,707	19,707	19,707	19,707	19,707	19,707
Model	probit	probit	probit	probit	probit	probit	probit	probit	probit	probit
**Dependent variable: Belonging to the specific labour market status**										
Explanatory variables:										
PGI	0.013	0.004	-0.009	-0.000	0.001	0.001	-0.003	-0.007	-0.006	0.004
	(0.015)	(0.010)	(0.018)	(0.013)	(0.014)	(0.010)	(0.016)	(0.010)	(0.016)	(0.011)
PGI squared		-0.001		0.010		-0.001		0.004		-0.008
		(0.007)		(0.009)		(0.007)		(0.007)		(0.008)
Female	-0.105***	-0.105***	-0.297***	-0.297***	-0.424***	-0.424***	0.657***	0.657***	-0.036	-0.036
	(0.021)	(0.021)	(0.026)	(0.026)	(0.021)	(0.021)	(0.020)	(0.020)	(0.021)	(0.021)
PGI # Female	-0.018		0.019		-0.000		-0.006		0.019	
	(0.021)		(0.026)		(0.020)		(0.020)		(0.021)	
Age	-0.256***	-0.256***	0.123***	0.123***	0.048***	0.048***	0.066***	0.066***	0.127***	0.127***
	(0.010)	(0.010)	(0.013)	(0.013)	(0.010)	(0.010)	(0.009)	(0.009)	(0.010)	(0.010)
Age squared	0.003***	0.003***	-0.001***	-0.001***	-0.001***	-0.001***	-0.001***	-0.001***	-0.002***	-0.002***
	(0.000)	(0.000)	(0.000)	(0.000)	(0.000)	(0.000)	(0.000)	(0.000)	(0.000)	(0.000)
PC1	-12.800***	-12.816***	-3.232	-3.249	-2.486	-2.484	2.275	2.260	16.551***	16.596***
	(1.506)	(1.507)	(1.811)	(1.811)	(1.438)	(1.438)	(1.365)	(1.365)	(1.478)	(1.478)
PC2	0.766	0.770	-0.747	-0.734	1.634	1.634	-0.655	-0.650	-1.666	-1.688
	(1.702)	(1.704)	(1.864)	(1.865)	(1.475)	(1.474)	(1.373)	(1.373)	(1.436)	(1.436)
PC3	4.010*	3.997*	-2.159	-2.153	-0.416	-0.416	0.200	0.193	-2.641	-2.624
	(1.480)	(1.480)	(1.805)	(1.805)	(1.452)	(1.451)	(1.316)	(1.316)	(1.386)	(1.386)
Year dummies	Yes	Yes	Yes	Yes	Yes	Yes	Yes	Yes	Yes	Yes
Birth cohort dummies	Yes	Yes	Yes	Yes	Yes	Yes	Yes	Yes	Yes	Yes
Constant	3.691***	3.693***	-3.904***	-3.920***	-1.168***	-1.167***	-1.764***	-1.768***	-3.542***	-3.534***
	(0.240)	(0.240)	(0.312)	(0.312)	(0.238)	(0.238)	(0.226)	(0.226)	(0.250)	(0.250)
Pseudo-R^2	0.128	0.128	0.027	0.027	0.034	0.034	0.067	0.067	0.035	0.035
p(χ^2)	***	***	***	***	***	***	***	***	***	***

** P < 0.05, ** P < 0.005, *** P < 0.001; robust SE in parentheses; SDPGI = polygenic index (PGI) for sleep duration (10)*

Table S14 Coefficient estimates from probit regression models of belonging to the knowledge work occupational group in the pooled 1992-2017 sample

**Sample**	**All**	**Males**	**Females**	**All**	**Males**	**Females**	**All**	**Males**	**Females**
**PGI**	**IPGI**	**IPGI**	**IPGI**	**SSPGI**	**SSPGI**	**SSPGI**	**LSPGI**	**LSPGI**	**LSPGI**
Model	probit	probit	probit	probit	probit	probit	probit	probit	probit
**Dependent variable: Belonging to the knowledge work occupational group**									
Explanatory variables:									
PGI Decile 1	Reference	Reference	Reference	Reference	Reference	Reference	Reference	Reference	Reference
PGI Decile 2	-0.108*	-0.043	-0.160*	-0.048	-0.012	-0.081	0.019	-0.057	0.100
	(0.047)	(0.069)	(0.064)	(0.047)	(0.068)	(0.064)	(0.047)	(0.068)	(0.066)
PGI Decile 3	-0.100*	-0.011	-0.174*	-0.127*	-0.118	-0.140*	0.028	-0.088	0.141*
	(0.047)	(0.069)	(0.063)	(0.048)	(0.070)	(0.065)	(0.047)	(0.068)	(0.066)
PGI Decile 4	-0.146**	-0.088	-0.195**	-0.133**	-0.161*	-0.109	-0.010	-0.054	0.038
	(0.047)	(0.070)	(0.063)	(0.047)	(0.070)	(0.064)	(0.048)	(0.067)	(0.067)
PGI Decile 5	-0.108*	-0.003	-0.197**	-0.116*	-0.115	-0.117	-0.026	-0.158*	0.103
	(0.047)	(0.069)	(0.064)	(0.047)	(0.070)	(0.064)	(0.048)	(0.069)	(0.066)
PGI Decile 6	-0.140**	-0.005	-0.255***	-0.142**	-0.190*	-0.105	-0.005	-0.069	0.063
	(0.047)	(0.069)	(0.064)	(0.048)	(0.071)	(0.065)	(0.047)	(0.067)	(0.067)
PGI Decile 7	-0.188***	-0.062	-0.292***	-0.094*	-0.088	-0.092	-0.095*	-0.178*	-0.008
	(0.047)	(0.069)	(0.065)	(0.047)	(0.069)	(0.065)	(0.048)	(0.068)	(0.067)
PGI Decile 8	-0.192***	-0.087	-0.282***	-0.152**	-0.137	-0.162*	-0.102*	-0.228**	0.021
	(0.047)	(0.069)	(0.065)	(0.048)	(0.071)	(0.064)	(0.048)	(0.070)	(0.066)
PGI Decile 9	-0.210***	-0.151*	-0.258***	-0.157***	-0.115	-0.196**	-0.084	-0.184*	0.018
	(0.047)	(0.070)	(0.064)	(0.047)	(0.070)	(0.064)	(0.048)	(0.069)	(0.067)
PGI Decile 10	-0.172***	-0.142*	-0.196**	-0.226***	-0.192*	-0.265***	-0.203***	-0.373***	-0.051
	(0.047)	(0.070)	(0.063)	(0.048)	(0.071)	(0.065)	(0.049)	(0.073)	(0.068)
Female	-0.037			-0.036			-0.033		
	(0.021)			(0.022)			(0.021)		
Age	0.128***	0.125***	0.133***	0.128***	0.125***	0.134***	0.128***	0.126***	0.134***
	(0.010)	(0.015)	(0.014)	(0.010)	(0.015)	(0.014)	(0.010)	(0.015)	(0.014)
Age squared	-0.002***	-0.001***	-0.002***	-0.002***	-0.001***	-0.002***	-0.002***	-0.001***	-0.002***
	(0.000)	(0.000)	(0.000)	(0.000)	(0.000)	(0.000)	(0.000)	(0.000)	(0.000)
’PC1	16.646***	19.173***	14.397***	16.974***	19.483***	14.602***	16.614***	18.991***	14.404***
	(1.472)	(2.149)	(2.028)	(1.478)	(2.157)	(2.034)	(1.471)	(2.148)	(2.025)
PC2	-1.538	0.231	-3.198	-1.451	0.248	-3.228	-1.543	0.379	-3.489
	(1.438)	(2.058)	(2.029)	(1.436)	(2.060)	(2.018)	(1.438)	(2.050)	(2.024)
PC3	-2.454	-1.132	-3.461	-2.433	-1.014	-3.629	-2.331	-0.927	-3.590
	(1.387)	(2.028)	(1.918)	(1.388)	(2.028)	(1.918)	(1.385)	(2.020)	(1.915)
Birth cohort dummies	Yes	Yes	Yes	Yes	Yes	Yes	Yes	Yes	Yes
Year dummies	Yes	Yes	Yes	Yes	Yes	Yes	Yes	Yes	Yes
Constant	-3.427***	-3.557***	-3.409***	-3.434***	-3.508***	-3.474***	-3.523***	-3.493***	-3.654***
	(0.252)	(0.368)	(0.346)	(0.252)	(0.368)	(0.346)	(0.252)	(0.368)	(0.346)
Pseudo-R^2	0.036	0.034	0.042	0.036	0.034	0.041	0.037	0.037	0.041
$p(\chi^2)$	***	***	***	***	***	***	***	***	***
N	19,707	9,068	10,639	19,707	9,068	10,639	19,707	9,068	10,639

** P < 0.05, ** P < 0.005, *** P < 0.001; robust SE in parentheses; polygenic indices (PGI) for insomnia (IPGI) (26), short sleep (SSPGI) (10), and long sleep (LSPGI) (10)*

Table S15 Differences in predicted probabilities of belonging to the knowledge work occupational group in the IPGI, SSPGI and LSPGI top three deciles compared to the lowest decile in the pooled 1992-2017 sample

**Sample**	**All**	**Males**	**Females**	**All**	**Males**	**Females**	**All**	**Males**	**Females**
**PGI**	**IPGI**	**IPGI**	**IPGI**	**SSPGI**	**SSPGI**	**SSPGI**	**LSPGI**	**LSPGI**	**LSPGI**
Total N in the model	19,707	9,068	10,639	19,707	9,068	10,639	19,707	9,068	10,639
**Reference: 1st decile PGI**									
Average marginal effects:									
**8th decile PGI**	-0.049***	-0.022	-0.072***	-0.039**	-0.036	-0.041*	-0.025*	-0.058**	0.005
	(0.012)	(0.018)	(0.016)	(0.012)	(0.018)	(0.016)	(0.012)	(0.018)	(0.015)
**9th decile PGI**	-0.053***	-0.037*	-0.067***	-0.040***	-0.030	-0.049**	-0.021	-0.048*	0.004
	(0.012)	(0.017)	(0.016)	(0.012)	(0.019)	(0.016)	(0.012)	(0.018)	(0.016)
**10th decile PGI**	-0.044***	-0.035*	-0.052**	-0.056***	-0.049*	-0.064***	-0.048***	-0.089***	-0.011
	(0.012)	(0.017)	(0.017)	(0.012)	(0.018)	(0.016)	(0.012)	(0.017)	(0.015)

** $P < 0.05$, ** $P < 0.005$, *** $P < 0.001$; robust SE in parentheses; average marginal effects from the models in SM Table 16; polygenic indices (PGI) for insomnia (IPGI) (26), short sleep (SSPGI) (10), and long sleep (LSPGI) (10)*

Table S16 Coefficient estimates from probit models of belonging to the knowledge work occupational group among those with higher education in the pooled 1992-2017 sample

**Sample**	**All with higher education**	**All with higher education**	**All with higher education**	**All with higher education**
**PGI**	**IPGI**	**SSPGI**	**LSPGI**	**SDPGI**
Model	probit	probit	probit	probit
N	7,456	7,456	7,456	7,456
**Dependent variable: Belonging to the knowledge work occupational group**				
Explanatory variables:				
PGI	-0.014	-0.047*	-0.067**	0.010
	(0.023)	(0.023)	(0.023)	(0.023)
Female	-0.254***	-0.251***	-0.246***	-0.252***
	(0.031)	(0.030)	(0.031)	(0.030)
PGI # Female	-0.021	0.025	0.059	-0.025
	(0.030)	(0.030)	(0.030)	(0.030)
Age	0.163***	0.163***	0.163***	0.163***
	(0.014)	(0.014)	(0.014)	(0.014)
Age squared	-0.002***	-0.002***	-0.002***	-0.002***
	(0.000)	(0.000)	(0.000)	(0.000)
PC1	9.998***	10.256***	9.863***	9.864***
	(2.057)	(2.063)	(2.056)	(2.064)
PC2	0.038	0.080	-0.058	0.013
	(2.071)	(2.067)	(2.071)	(2.074)
PC3	-3.171	-3.124	-3.180	-3.286
	(2.035)	(2.034)	(2.032)	(2.033)
Year dummies	Yes	Yes	Yes	Yes
Birth cohort dummies	Yes	Yes	Yes	Yes
Constant	-3.482***	-3.493***	-3.485***	-3.483***
	(0.350)	(0.350)	(0.350)	(0.350)
Pseudo-R^2	0.030	0.030	0.030	0.029
$p(\chi^2)$	***	***	***	***

** P < 0.05, ** P < 0.005, *** P < 0.001; robust SE in parentheses; polygenic indices (PGI) for insomnia (IPGI) (26), short sleep (SSPGI) (10), long sleep (LSPGI) (10), and sleep duration (SDPGI) (10)*

Table S17 Coefficient estimates from ordered probit models of income quintiles in the pooled 1992-2017 sample

**Sample**	**All**	**Males**	**Females**	**All**	**Males**	**Females**	**All**	**Males**	**Females**
**PGI**	**IPGI**	**IPGI**	**IPGI**	**SSPGI**	**SSPGI**	**SSPGI**	**LSPGI**	**LSPGI**	**LSPGI**
Model	oprobit	oprobit	oprobit	oprobit	oprobit	oprobit	oprobit	oprobit	oprobit
N	19,685	9,098	10,587	19,685	9,098	10,587	19,685	9,098	10,587
**Dependent variable: Income quintile**									
Explanatory variables:									
PGI	-0.036**	-0.035**	-0.034***	-0.037**	-0.037***	-0.041***	-0.037**	-0.036**	-0.023*
	(0.012)	(0.011)	(0.010)	(0.011)	(0.011)	(0.010)	(0.011)	(0.011)	(0.010)
Female	-0.059***			-0.058***			-0.057***		
	(0.015)			(0.015)			(0.015)		
PGI # Female	0.002			-0.004			0.013		
	(0.015)			(0.015)			(0.015)		
Age	0.167***	0.179***	0.158***	0.167***	0.179***	0.159***	0.167***	0.179***	0.159***
	(0.007)	(0.011)	(0.010)	(0.007)	(0.011)	(0.010)	(0.007)	(0.011)	(0.010)
Age squared	-0.002***	-0.002***	-0.002***	-0.002***	-0.002***	-0.002***	-0.002***	-0.002***	-0.002***
	(0.000)	(0.000)	(0.000)	(0.000)	(0.000)	(0.000)	(0.000)	(0.000)	(0.000)
PC1	13.813***	15.234***	12.419***	14.111***	15.601***	12.658***	13.757***	15.188***	12.354***
	(1.106)	(1.603)	(1.531)	(1.107)	(1.603)	(1.533)	(1.107)	(1.603)	(1.533)
PC2	-2.846*	-2.914	-2.766	-2.762*	-2.801	-2.709	-2.890*	-2.909	-2.854
	(1.234)	(1.829)	(1.685)	(1.235)	(1.832)	(1.684)	(1.238)	(1.836)	(1.691)
PC3	-3.083**	-4.037*	-2.392	-3.071**	-3.992*	-2.405	-3.058**	-3.999*	-2.380
	(1.088)	(1.610)	(1.478)	(1.087)	(1.610)	(1.476)	(1.088)	(1.608)	(1.478)
Year dummies	Yes	Yes	Yes	Yes	Yes	Yes	Yes	Yes	Yes
Birth cohort dummies	Yes	Yes	Yes	Yes	Yes	Yes	Yes	Yes	Yes
Cut: income quintiles 1/2	2.418***	2.850***	2.132***	2.423***	2.859***	2.134***	2.423***	2.841***	2.146***
	(0.174)	(0.258)	(0.235)	(0.174)	(0.258)	(0.235)	(0.174)	(0.258)	(0.235)
Cut: income quintiles 2/3	3.020***	3.440***	2.746***	3.026***	3.448***	2.748***	3.025***	3.431***	2.760***
	(0.174)	(0.258)	(0.235)	(0.174)	(0.259)	(0.235)	(0.174)	(0.259)	(0.235)
Cut: income quintiles 3/4	3.527***	3.913***	3.281***	3.532***	3.922***	3.283***	3.531***	3.904***	3.295***
	(0.174)	(0.259)	(0.236)	(0.174)	(0.259)	(0.236)	(0.174)	(0.259)	(0.236)
Cut: income quintiles 4/5	4.135***	4.512***	3.899***	4.140***	4.521***	3.900***	4.139***	4.503***	3.912***
	(0.175)	(0.260)	(0.236)	(0.175)	(0.260)	(0.236)	(0.175)	(0.260)	(0.236)
Pseudo-R^2	0.024	0.024	0.024	0.024	0.024	0.024	0.024	0.024	0.024
$p(\chi^2)$	***	***	***	***	***	***	***	***	***

** P < 0.05, ** P < 0.005, *** P < 0.001; robust SE in parentheses; polygenic indices (PGI) for insomnia (IPGI) (26), short sleep (SSPGI) (10), and long sleep (LSPGI) (10)*

Table S18 Coefficient estimates from ordered probit models of income quintiles among those with higher education in the pooled 1992-2017 sample

**Sample**	**All with higher education**	**Males with higher education**	**Females with higher education**	**All with higher education**	**Males with higher education**	**Females with higher education**	**All with higher education**	**Males with higher education**	**Females with higher education**
**PGI**	**IPGI**	**IPGI**	**IPGI**	**SSPGI**	**SSPGI**	**SSPGI**	**LSPGI**	**LSPGI**	**LSPGI**
Model	oprobit	oprobit	oprobit	oprobit	oprobit	oprobit	oprobit	oprobit	oprobit
N	7,498	3,028	4,470	7,498	3,028	4,470	7,498	3,028	4,470
**Dependent variable: Income quintile**									
Explanatory variables:									
PGI	-0.012	-0.012	-0.023	-0.037	-0.037	-0.032*	-0.043*	-0.043*	0.004
	(0.021)	(0.020)	(0.016)	(0.020)	(0.020)	(0.016)	(0.020)	(0.020)	(0.016)
Female	-0.146***			-0.145***			-0.142***		
	(0.026)			(0.026)			(0.026)		
PGI # Female	-0.010			0.007			0.045		
	(0.026)			(0.026)			(0.026)		
Age	0.195***	0.217***	0.188***	0.195***	0.218***	0.188***	0.195***	0.216***	0.188***
	(0.012)	(0.019)	(0.015)	(0.012)	(0.020)	(0.015)	(0.012)	(0.019)	(0.015)
Age squared	-0.002***	-0.002***	-0.002***	-0.002***	-0.002***	-0.002***	-0.002***	-0.002***	-0.002***
	(0.000)	(0.000)	(0.000)	(0.000)	(0.000)	(0.000)	(0.000)	(0.000)	(0.000)
PC1	8.916***	9.666***	8.693***	9.212***	10.133***	8.895***	8.838***	9.630***	8.594***
	(1.785)	(2.803)	(2.333)	(1.785)	(2.796)	(2.335)	(1.785)	(2.804)	(2.332)
PC2	-3.032	-2.702	-3.045	-2.981	-2.666	-2.974	-3.114	-2.831	-3.076
	(1.949)	(2.886)	(2.680)	(1.942)	(2.864)	(2.677)	(1.952)	(2.882)	(2.685)
PC3	-1.220	-0.528	-1.832	-1.114	-0.275	-1.821	-1.245	-0.385	-1.983
	(1.808)	(2.917)	(2.323)	(1.807)	(2.918)	(2.322)	(1.805)	(2.914)	(2.319)
Year dummies	Yes	Yes	Yes	Yes	Yes	Yes	Yes	Yes	Yes
Birth cohort dummies	Yes	Yes	Yes	Yes	Yes	Yes	Yes	Yes	Yes
Cut: income quintiles 1/2	2.634***	3.393***	2.457***	2.641***	3.424***	2.447***	2.631***	3.381***	2.453***
	(0.285)	(0.477)	(0.356)	(0.285)	(0.478)	(0.356)	(0.285)	(0.476)	(0.356)
Cut: income quintiles 2/3	3.234***	3.970***	3.073***	3.242***	4.002***	3.064***	3.232***	3.959***	3.070***
	(0.286)	(0.478)	(0.357)	(0.286)	(0.479)	(0.357)	(0.286)	(0.478)	(0.357)
Cut: income quintiles 3/4	3.743***	4.427***	3.616***	3.750***	4.459***	3.606***	3.741***	4.416***	3.612***
	(0.287)	(0.480)	(0.358)	(0.287)	(0.480)	(0.358)	(0.287)	(0.479)	(0.358)
Cut: income quintiles 4/5	4.410***	5.093***	4.287***	4.418***	5.125***	4.278***	4.408***	5.082***	4.283***
	(0.287)	(0.481)	(0.359)	(0.288)	(0.482)	(0.359)	(0.287)	(0.481)	(0.359)
Pseudo-R^2	0.031	0.027	0.031	0.031	0.028	0.031	0.031	0.028	0.031
$p(\chi^2)$	***	***	***	***	***	***	***	***	***

** P < 0.05, ** P < 0.005, *** P < 0.001; robust SE in parentheses; polygenic indices (PGI) for insomnia (IPGI) (26), short sleep (SSPGI) (10), and long sleep (LSPGI) (10)*

Table S19 Coefficient estimates from ordered probit models of income quintiles in the pooled 1992-2017 sample of employees with higher education in the knowledge work occupational group

**Sample**	**All**	**Males**	**Females**	**All**	**Males**	**Females**	**All**	**Males**	**Females**
	**Employees with higher education in the knowledge work occupational group**								
**PGI**	**IPGI**	**IPGI**	**IPGI**	**SSPGI**	**SSPGI**	**SSPGI**	**LSPGI**	**LSPGI**	**LSPGI**
Model	oprobit	oprobit	oprobit	oprobit	oprobit	oprobit	oprobit	oprobit	oprobit
N	2,910	1,354	1,556	2,910	1,354	1,556	2,910	1,354	1,556
**Dependent variable: Income quintile**									
Explanatory variables:									
PGI	0.023	0.019	0.004	-0.010	-0.016	-0.045	-0.019	-0.022	-0.011
	(0.032)	(0.033)	(0.027)	(0.031)	(0.032)	(0.030)	(0.032)	(0.033)	(0.029)
Female	-0.170***			-0.170***			-0.167***		
	(0.043)			(0.043)			(0.043)		
PGI # Female	-0.022			-0.036			0.006		
	(0.042)			(0.044)			(0.044)		
Age	0.129***	0.153***	0.113***	0.130***	0.154***	0.114***	0.130***	0.155***	0.113***
	(0.022)	(0.034)	(0.030)	(0.022)	(0.034)	(0.030)	(0.022)	(0.034)	(0.030)
Age squared	-0.001***	-0.001***	-0.001**	-0.001***	-0.001***	-0.001**	-0.001***	-0.001***	-0.001**
	(0.000)	(0.000)	(0.000)	(0.000)	(0.000)	(0.000)	(0.000)	(0.000)	(0.000)
PC1	11.765***	16.202***	8.732*	12.006***	16.443***	9.055*	11.800***	16.260***	8.760*
	(3.085)	(4.661)	(4.163)	(3.088)	(4.650)	(4.179)	(3.088)	(4.656)	(4.172)
PC2	-3.035	-1.189	-4.740	-2.900	-1.187	-4.454	-3.059	-1.247	-4.730
	(3.193)	(4.992)	(4.329)	(3.179)	(4.926)	(4.337)	(3.182)	(4.966)	(4.333)
PC3	1.693	6.343	-1.872	1.811	6.515	-1.769	1.805	6.438	-1.747
	(2.988)	(4.900)	(3.807)	(2.988)	(4.901)	(3.807)	(2.980)	(4.895)	(3.793)
Year dummies	Yes	Yes	Yes	Yes	Yes	Yes	Yes	Yes	Yes
Birth cohort dummies	Yes	Yes	Yes	Yes	Yes	Yes	Yes	Yes	Yes
Cut: income quintiles 1/2	1.095*	1.635*	0.909	1.110*	1.665*	0.914	1.113*	1.671*	0.909
	(0.531)	(0.809)	(0.709)	(0.531)	(0.810)	(0.708)	(0.531)	(0.808)	(0.709)
Cut: income quintiles 2/3	1.764***	2.268*	1.604*	1.780***	2.298**	1.611*	1.782***	2.304**	1.604*
	(0.531)	(0.813)	(0.708)	(0.531)	(0.814)	(0.707)	(0.531)	(0.812)	(0.707)
Cut: income quintiles 3/4	2.291***	2.810***	2.126**	2.307***	2.840***	2.134**	2.308***	2.845***	2.126**
	(0.532)	(0.813)	(0.709)	(0.532)	(0.814)	(0.708)	(0.531)	(0.813)	(0.709)
Cut: income quintiles 4/5	3.011***	3.604***	2.795***	3.028***	3.633***	2.803***	3.029***	3.639***	2.795***
	(0.532)	(0.813)	(0.711)	(0.532)	(0.814)	(0.709)	(0.532)	(0.813)	(0.710)
Pseudo-R^2	0.041	0.043	0.039	0.041	0.043	0.039	0.041	0.043	0.039
p(χ^2)	***	***	***	***	***	***	***	***	***

** P < 0.05, ** P < 0.005, *** P < 0.001; robust SE in parentheses; polygenic indices (PGI) for insomnia (IPGI) (26), short sleep (SSPGI) (10), and long sleep (LSPGI) (10)*

Table S20 Coefficient estimates from ordered probit models of income quintiles in the pooled 1992-2017 sample

**Sample**	**All**	**All**	**Males**	**Females**
**PGI**	**SDPGI linear**	**SDPGI linear + squared**	**SDPGI linear**	**SDPGI linear**
Model	oprobit	oprobit	oprobit	oprobit
N	19,685	19,685	9,098	10,587
**Dependent variable: Income quintile**				
Explanatory variables:				
SDPGI	0.008	0.011	0.009	0.012
	(0.011)	(0.008)	(0.011)	(0.010)
SDPGI squared		-0.002		
		(0.005)		
Female	-0.059***	-0.059***		
	(0.015)	(0.015)		
SDPGI # Female	0.004			
	(0.015)			
Age	0.167***	0.167***	0.179***	0.158***
	(0.007)	(0.007)	(0.011)	(0.010)
Age squared	-0.002***	-0.002***	-0.002***	-0.002***
	(0.000)	(0.000)	(0.000)	(0.000)
PC1	13.863***	13.874***	15.327***	12.437***
	(1.110)	(1.110)	(1.609)	(1.536)
PC2	-2.901*	-2.903*	-2.939	-2.847
	(1.240)	(1.240)	(1.842)	(1.689)
PC3	-3.213**	-3.210**	-4.130*	-2.553
	(1.088)	(1.088)	(1.611)	(1.477)
Year dummies	Yes	Yes	Yes	Yes
Birth cohort dummies	Yes	Yes	Yes	Yes
Cut: income quintiles 1/2	2.414***	2.412***	2.841***	2.132***
	(0.174)	(0.174)	(0.258)	(0.235)
Cut: income quintiles 2/3	3.016***	3.013***	3.430***	2.746***
	(0.174)	(0.174)	(0.259)	(0.235)
Cut: income quintiles 3/4	3.522***	3.519***	3.903***	3.281***
	(0.174)	(0.175)	(0.259)	(0.236)
Cut: income quintiles 4/5	4.129***	4.127***	4.502***	3.898***
	(0.175)	(0.175)	(0.260)	(0.236)
Pseudo-R^2	0.023	0.023	0.023	0.024
$p(\chi^2)$	***	***	***	***

** P < 0.05, ** P < 0.005, *** P < 0.001; robust SE in parentheses; SDPGI = polygenic index for sleep duration (10)*
